# Influence of environmental parameters on movements and habitat utilization of humpback whales (*Megaptera novaeangliae*) in the Madagascar breeding ground

**DOI:** 10.1098/rsos.160616

**Published:** 2016-12-21

**Authors:** Laurène Trudelle, Salvatore Cerchio, Alexandre N. Zerbini, Ygor Geyer, François-Xavier Mayer, Jean-Luc Jung, Maxime R. Hervé, Stephane Pous, Jean-Baptiste Sallée, Howard C. Rosenbaum, Olivier Adam, Jean-Benoit Charrassin

**Affiliations:** 1 Bioacoustics Team, Institut de Neurosciences Paris-Saclay (NeuroPSI), CNRS UMR 8195, Université Paris Sud, 91405 Orsay, France; 2 Sorbonne Universités (UPMC, Univ. Paris 06)—CNRS-IRD-MNHN, LOCEAN-IPSL, 4 place Jussieu, 75005 Paris, France; 3 Biotope, Unité Recherche et Développement, 22 Boulevard Maréchal Foch, BP 58, 34140 Meze, France; 4 New England Aquarium, Central Wharf, Boston, MA, USA; 5 Woods Hole Oceanographic Institution, 266 Woods Hole Road, Woods Hole, MA 02543, USA; 6 National Marine Mammal Laboratory, Alaska Fisheries Science Center, National Marine Fisheries Service, NOAA, 7600 Sand Point Way NE, Seattle, WA 98125, USA; 7 Cascadia Research Collective, 218 1/2 4th Avenue W, Olympia, WA 98501, USA; 8 Instituto Aqualie, Av. Dr. Paulo Japiassú 714/206, Juiz de Fora, MG 36033-310, Brazil; 9 Cetamada, Port Barachois, Ambodifotatra BP 5, 515 Sainte Marie, Madagascar; 10 BioGemme Laboratory, Brest, France; 11 Institute of Plant Sciences—‘Biotic interactions’ group, Altenbergrain 21, 3013 Bern, Switzerland; 12 INRA, UMR1349 IGEPP, 35653 Le Rheu, France; 13 Department of Oceanography, University of Cape Town, Cape Town, South Africa; 14 Ocean Giants Program, Wildlife Conservation Society, 2300 Southern Blvd, Bronx, NY 10460, USA; 15 Sorbonne Université, UPMC Univ Paris 06, CNRS UMR 7190, Institut Jean Le Rond d'Alembert, 75005 Paris, France

**Keywords:** humpback whales, satellite telemetry, Madagascar, movement patterns, environmental parameters, habitat use

## Abstract

Assessing the movement patterns and key habitat features of breeding humpback whales is a prerequisite for the conservation management of this philopatric species. To investigate the interactions between humpback whale movements and environmental conditions off Madagascar, we deployed 25 satellite tags in the northeast and southwest coast of Madagascar. For each recorded position, we collated estimates of environmental variables and computed two behavioural metrics: behavioural state of ‘transiting’ (consistent/directional) versus ‘localized’ (variable/non-directional), and active swimming speed (i.e. speed relative to the current). On coastal habitats (i.e. bathymetry < 200 m and in adjacent areas), females showed localized behaviour in deep waters (191 ± 20 m) and at large distances (14 ± 0.6 km) from shore, suggesting that their breeding habitat extends beyond the shallowest waters available close to the coastline. Males' active swimming speed decreased in shallow waters, but environmental parameters did not influence their likelihood to exhibit localized movements, which was probably dominated by social factors instead. In oceanic habitats, both males and females showed localized behaviours in shallow waters and favoured high chlorophyll-*a* concentrations. Active swimming speed accounts for a large proportion of observed movement speed; however, breeding humpback whales probably exploit prevailing ocean currents to maximize displacement. This study provides evidence that coastal areas, generally subject to strong human pressure, remain the core habitat of humpback whales off Madagascar. Our results expand the knowledge of humpback whale habitat use in oceanic habitat and response to variability of environmental factors such as oceanic current and chlorophyll level.

## 1. Introduction

Most baleen whales are highly mobile, and their distribution and abundance are influenced by the marine environment at different spatial and temporal scales [1]. Migratory species, such as the humpback whale (*Megaptera novaeangliae*), have a broad spatial range and therefore can exploit a variety of habitats (e.g. feeding versus breeding grounds in the case of migratory baleen whales). Distribution and habitat preferences over the annual cycle are partially dependent on environmental parameters, including oceanographic and climatological characteristics [2]. While the most frequently invoked factor to explain movement and habitat patterns in animals is resource availability [3], other factors such as predation risk or social interaction with conspecifics can affect both temporal distribution and habitat selection [4–6]. Habitat heterogeneity, biological requirements and social behaviour of a given species interact to influence patterns of distribution and habitat use.

The migratory cycle of humpback whales alternates seasonally between a summer feeding period in productive high latitudes and a winter breeding period in low latitudes [7,8]. Humpback whales typically fast during the breeding period, because productivity in wintering habitat is generally low. One explanation for why humpback whales migrate is to reduce the predation threat on their breeding grounds [9]. Other hypotheses have been suggested such as energetic advantages (e.g. warm water temperatures) which could benefit calf growth and reproductive success, but this is still debated[10–12]. The aggregation of humpback whales in localized breeding areas is a consequence of their mating system which involves intrasexual competition among males for females [8,13,14]. Furthermore, the widespread distribution of females is assumed to result from the absence of predation and prey [8]. In this context, social organization, breeding status or environmental variables seem to shape the selection of habitat [15,16].

Studies on different humpback whale breeding areas around the world show that animals occur in high densities in a variety of habitats, including continental coasts, coastal and oceanic islands, reef systems or over shoals (e.g. [16–20]). Humpback whales are frequently found in warm (21.1–28°C), shallow (15–60 m depth), near-shore or shelf waters [7,12,21–23]. In particular, it is believed that females with calves tend to prefer shallow waters in protected areas (20 m depth or less) [16,24]. Social groups, as mother–calf pairs, could also show a flexible response and adopt alternative behavioural strategies according to abiotic features of regions and the potential human disturbance [25]. Typically, humpback whales are found within close proximity to the coast during the breeding period. However, they are also found in deep waters [26,27] and in shallow waters extended offshore during winter [28,29], where movement patterns and habitat use are poorly known. To date, only one study has investigated the movements of individual humpback whales in a breeding ground and established direct links with environmental parameters [30].

The range of the southwestern Indian Ocean humpback whale population, referred to as Breeding Stock C by the International Whaling Commission (IWC), extends from southeastern Africa to the Mascarenes Islands [31,32]. Within this region, Madagascar Island, defined as the subregion C3, is an important breeding ground for humpback whales [33–40]. The population abundance of humpback whales around Madagascar in 2015 was estimated at 8854 (95% CI of 6906–16 106) whales [39,41]. Humpback whales are widely distributed around Madagascar [33,34,36,42,43], but higher concentrations are reported in certain areas including the northeast (Ile Sainte Marie, Antongil bay) [15,39,44,45], and the southern region from Toliara to Fort Dauphin [39,46].

While the western coast of Madagascar is characterized by a wide continental shelf and an extensive barrier reef (90 km at its widest, approx. 16° S; [47]), the east coast features a narrow continental shelf of 25–50 km width and an abrupt break in the slope below 1000 m [48]. The northeastern coast is where the continental shelf is the widest, with Antongil Bay and Ile Sainte Marie characterized by extensive shallow waters [15]. The southern tip of Madagascar is also characterized by a wide continental shelf extending 50 km from land and is referred to as the Madagascar Plateau [49]. Sightings of humpback whales have been recorded further south than the southern tip of Madagascar around the seamount of Walters Shoals located 645 km south of Madagascar and at depths less than 20 m [31,50].

The strongest geostrophic currents (0.2–1 m.s) are observed on the east coast of Madagascar, up to the south of Ile Sainte Marie and Antongil Bay region (electronic supplementary material, figure S1, reconstructed from the dataset of SWIO12 climatological model (see *environmental parameters*))*.* The South Equatorial Current (SEC), which flows westward at around 20° S, splits into two branches as it approaches the eastern Madagascar coast: one branch flows northward to the northern tip of Madagascar; and the other flows southward along Madagascar, referred to as the East Madagascar Current (EMC) [51,52] (electronic supplementary material, figure S1). The ocean circulation of the west coast is poorly known, and is defined by slower currents and strong mesoscale features (i.e. eddies) southwest of Madagascar [53,54]. Whether and how these contrasting oceanographic characteristics affect humpback whale habitat utilization around Madagascar during the breeding period has not been investigated to this point (electronic supplementary material, figure S1).

The biology of humpback whales is one of the best known among large whales, but our knowledge of individual movement patterns and habitat use in offshore habitats (i.e. far away from the shoreline along continental shelves and open-ocean habitats) during the breeding season remains limited and probably biased due to a lack of data in regions that are logistically challenging to sample [20,26,55]. Recent technological improvements in cetacean satellite telemetry have increased information on seasonal distribution ranges, movements into remote areas, as well as migration route and stock structure [26,27,56–58]. It is also now possible to relate the individual whale's horizontal movements to their physical environment at appropriate spatial and temporal scales, and to better understand how environmental parameters influence whales' distribution.

In this study, we used data from Argos satellite tags attached to humpback whales off Ile Sainte Marie (northeast Madagascar) and Anakao (southwest Madagascar) during three breeding seasons to investigate movement patterns and habitat utilization of humpback whales in relation to sex and reproductive status. We then investigated how key environmental variables along the whale tracks influenced humpback whale movements.

Identified as important variables in previous published studies investigating the relationship between humpback whale distribution and the environment, six environmental variables were selected including: (1) sea surface temperature (SST), (2) seabed depth (BAT), (3) sea floor slope (SL), (4) distance from shore (DIST), (5) ocean current speed (SSC) and direction, and (6) surface chlorophyll-*a* concentration (CHL). Because the basin scale, the distribution of humpback whale breeding grounds, is defined by a relatively narrow SST range, we examined whether SST may influence whale movements and habitat preferences at a regional scale. Humpback whales are frequently seen on continental shelves, in shallow waters and reef areas during the breeding season, suggesting that BAT and SL could be important drivers of their distribution. SSC has also been investigated as it is likely to influence the whale's heading and active swimming speed. Finally, CHL is used as a proxy for the marine ecosystem productivity, in order to investigate if whales could feed opportunistically in favourable areas in tropical waters (e.g. thermal front, seamount). Although it is clear that humpback whales fast during winter, some occasional feeding events have been documented in winter populations (e.g. [59–61]).

## 2. Material and methods

### 2.1. Tag deployment

Twenty-five humpback whales were equipped with Wildlife Computer SPOT 5 satellite transmitters during the breeding season over the years 2012–2014 (table 1; hereafter we will refer to individual whales using their numerical ID as shown in that table). Whales were tagged in two distinct regions of Madagascar: Ile Sainte Marie (16°50′ S, 49°55′ E) in 2012 and 2014, and off Anakao (23°40′ S, 43°39′ E) in 2013. They were selected for tagging based on sex, reproductive class and behavioural subclass. The latter category includes: single individual (SOLO), one of two individuals in a pair (Pair), an animal in a competitive group with an undetermined role (CG), a principal escort (PE), a secondary escort (SE), a challenger (CH), a nuclear animal (NA), a female with a calf of the year (Mom) and escort to a mother (Esc) (see details in [62]). The sex of each tagged individual was inferred in the field based upon the presence of a calf (i.e. assuming that adults in cow–calf pairs were females), the role in the group of whales (see [62]), when possible, and except for one whale was subsequently confirmed through genetic analysis from biopsy samples [63]. Whale locations were obtained through the Argos data collection system [64]. Further details about tag anchoring, deployment and whale selection can be found in [62]. In 2012, we used both an 8 m long carbon fibre pole, and modified pneumatic tag deliverer (ARTS) to deploy tags on whales, and in subsequent years, we only used the ARTS [26,27,57]. Tags deployed in 2012 were duty-cycled to transmit 6 h on, 6 h off for the first three months after deployment, and then every other day until the end of transmission. In 2013, tags were duty-cycled to transmit 9 h on, 3 h off to increase data collection. In 2014, tags were duty-cycled to transmit 2 h on 5 h off during the day, and 2 h on 4 h off during the night. Without a long focal follow of a whale after tagging, it is impossible to know how long the tag disturbance lasted. During the focal follow period (approx. 10 min), responses of the whale to the tagging varied from none to short-term disturbance. In all cases in which the whale was actively engaged in a specific behaviour (e.g. within a competitive group), the whale continued with that behaviour pattern after a brief reaction to the immediate tagging event. No whale exhibited obvious signs of severe disturbance (e.g. leaving a group, flight or repeated percussive surface activity). As first whale positions are transmitted beyond 1 h after tagging; all data after tagging were used and considered undisturbed behaviour.

**Table 1.** Summary of the tracking dataset of humpback whales equipped at Sainte Marie channel (SM) and Anakao (AK) and main characteristics of breeding movements based on switching state space model (SSSM) positions estimated every 12 h. Group types include singleton, S; pair, P; competitive group, CG; non-competitive group, NCG; mother--calf pair, MC; mother--calf escort, MCE; mother--calf with more than one escort, MCES. Whale's tracks are defined by coastal (C) or/and oceanic (O) movements. Means are expressed ± s.e. Asterisks indicate that mean values were computed on all location values, whereas mean values used in statistical tests were computed by individual.

whale id	tag location	sex	group type	tag date	tag longevity (days)	number of location data points	number of estimated locations after application of SSSM	type of movements	travelled distance per day (km)	b-mode average	observed swimming speed (m s^−1^)
1	SM	M	S	24 July 2012	32	123				—	—
					3	—		—		—	—
					13	—	27	C, O	47 ± 6.88	1.26	1.02 ± 0.12
2	SM	M	CG	30 July 2012	31	231	62	C	22 ± 2.4	1.88	0.46 ± 0.03
3	SM	M	P	30 July 2012	20	169	41	C	29 ± 2.97	1.63	0.62 ± 0.09
4	SM	F	MC	31 July 2012	25	58				—	—
					8	—	17	C	34 ± 8.6	1.44	0.72 ± 0.17
					4	—		—		—	—
5	SM	M	MCE	01 August 2012	5	21		—		—	—
6	SM	M	P	31 July 2012	10	71	20	C	31 ± 4.4	1.79	0.65 ± 0.09
7	SM	F	MC	31 July 2012	13	102	27	C, O	38 ± 3.6	1.05	0.85 ± 0.09
8	SM	M	CG	01 August 2012	3	21		—		—	—
9	SM	F	CG	01 August 2012	58	368	116	C, P	44 ± 2.8	1.19	1 ± 0.06
10	SM	F	MC	01 August 2012	30	222	61	C	29 ± 3.2	1.32	0.6 ± 0.06
11	SM	M	CG	03 August 2012	15	104	30	C	28 ± 4.9	1.65	0.6 ± 0.12
12	SM	F	CG	03 August 2012	23	196	46	C, O	43 ± 4.8	1.05	0.9 ± 0.09
13	AK	unknown	NCG	16 July 2013	15	206	31	C, O	37 ± 3.6	1.25	0.86 ± 0.09
14	AK	F	P	17 July 2013	21	8		—		—	—
15	AK	F	P	17 July 2013	42	473	85	C	37. ± 3.1	1.17	0.8 ± 0.06
16	AK	F	P	17 July 2013	2	13		—		—	—
17	AK	M	P	17 July 2013	34	480	68	C, O	30 ± 2.6	1.24	0.71 ± 0.73
18	AK	M	CG	21 July 2013	23	307	45	C	30 ± 2.5	1.57	0.7 ± 0.06
19	AK	M	CG	21 July 2013	8	85	10	C	54 ± 9.3	1.39	0.96 ± 0.12
20	AK	F	MBE	23 July 2013	23	269	36	C	44 ± 5.7	1.03	0.83 ± 0.06
21	AK	F	CG	25 July 2013	17	129	34	C	28 ± 3.9	1.31	0.6 ± 0.06
22	AK	F	NCG	27 July 2013	56	786	105	C, O	32 ± 1.9	1.24	0.74 ± 0.06
23	AK	F	MC	28 July 2013	52	807	104	C	22 ± 1.8	1.17	0.48 ± 0.03
24	SM	M	MCES	21 August 2014	4	15		—		—	—
25	SM	F	MC	23 August 2014	37	367	74	C	23 ± 2.06	1.36	0.52 ± 0.06
								C		1.34 ± 0.01*	0.8 ± 0.02*
								O		1.14 ± 0.01*	1.12 ± 0.05*
								O (whale 22 not included)		1.17 ± 0.02*	1.21 ± 0.08*

### 2.2. Data processing

All locations classified invalid by Argos (Argos location class ‘Z’) and all locations on land were removed from the dataset. We retained Argos locations, classes 3, 2, 1, 0, A and B (see http://www.argos-system.org/manual/3-location/34_location_classes.htm), and assumed as invalid any location implying a travel rate greater than 12 km h^−1^ (e.g. [19]) as indicated by R Package Trip (R Development Core Team 2006). The Bayesian Switching State Space Model (SSSM) was used to model estimated positions and associated errors based upon the raw Argos locations [65] to provide the best estimate of each whale's path. A 12 h time step was used to minimize the number of positions estimated when the tags were not transmitting. The model was fitted using WinBUGS v. 1.4. Two Markov Chain Monte Carlo (MCMC) chains were run in parallel, each for a total of 50 000 simulations. The first 20 000 samples were discarded as a ‘burn-in’ and the remaining samples were thinned, retaining every 30th sample to reduce autocorrelation. The 1000 retained iterations for each chain, giving a total of 2000 independent samples, were used to compute the posterior distribution of the parameters of interest, including behavioural mode. The behavioural mode parameter measures the likelihood of exhibiting localized movements based on the mean turning angle, and the autocorrelation of speed and direction into the first difference correlated random walk model within the SSSM [66]. It is a behaviour index estimated at each location by the SSSM. A b-mode approaching 1 means a highly directional and consistent long-distance movement that represents a ‘transiting’ behaviour. For example, animals tend to travel between/through breeding areas or migrate. A b-mode approaching 2 means more variable movements and marked changes in direction that represent a ‘localized’ behaviour. For example, animals are engaged in breeding activities and meandering movements within breeding habitat. A behavioural mode less than 1.25 was considered as an indication of a transiting behaviour [65]. Details on estimation procedure are presented in Jonsen *et al.* [66,67].

To avoid introduction of unrepresentative estimated locations or behavioural state values, all tracks shorter than 8 days were excluded from analysis (whales 5, 8, 14, 16, 24; table 1). For tracks showing long transmission gaps (e.g. 16 days and 12 days for whales 1 and 4, respectively; table 1), tracks were split into two segments that were analysed separately. The first segment of whale 1 and the second segment of whale 4 were excluded from subsequent analyses as they were less than 8 days of data.

### 2.3. Data organization

**Figure 1.**
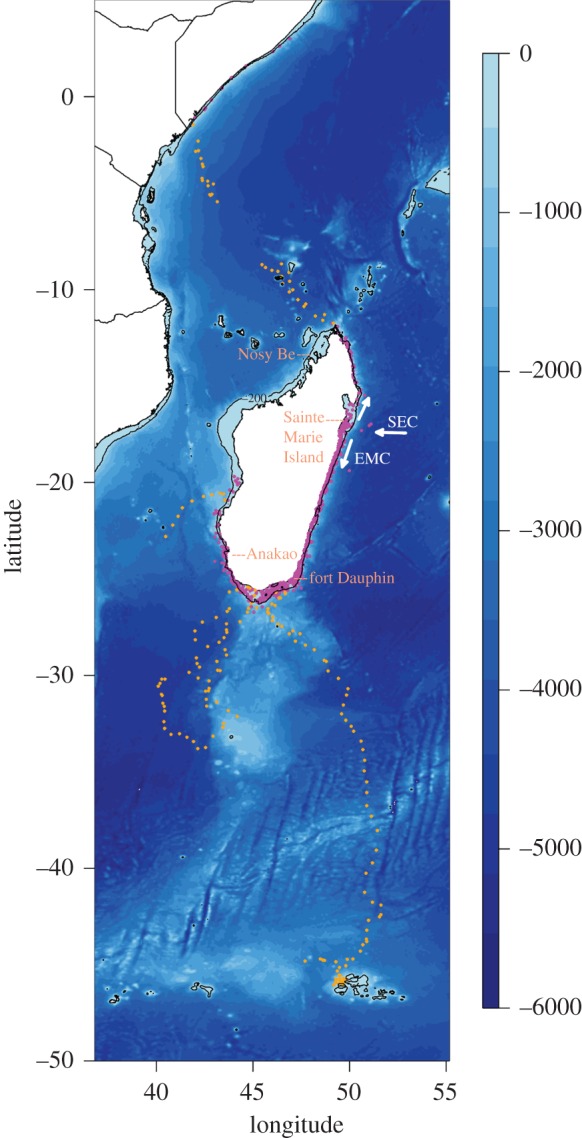
Whale locations associated with the type of movements (purple = coastal, orange = oceanic). Arrows illustrate the South Equatorial Current (SEC), and the southern branch of the SEC, known as the East Madagascar Current (EMC).

The whale position data were split into two categories: coastal and oceanic according to whale position relative to the bathymetric environment from which the whales evolved (figure 1). Whale movements were defined as coastal when located over the continental shelf (less than 200 m water depth, 78% of coastal positions) or around the shelf break (more than 200 m water depth, 22% of coastal positions). Whale movements were defined as oceanic when animals were definitively leaving the Madagascar continental shelf towards deep ocean waters (i.e. all whale locations that were over a depth more than 200 m and not associated with coastal movements).

### 2.4. Environmental parameters

Three static variables (BAT, SL and DIST) and three dynamic variables (CHL, SST and SSC) were estimated at each whale time and position. The GEBCO bathymetry Grid-database (30 s per cell, http://www.gebco.net/) was used to compute bathymetry depth, bathymetry slope and DIST. Sea floor slope (hereafter ‘slope’) was derived using the R package raster (function *terrain*; [68]). The whale DIST was calculated as the distance between each location and the nearest coastline defined by a positive bathymetry.

CHL was estimated from the merged daily GlobColour product at a 4 km (0.1°) spatial resolution (http://www.globcolour.info/). We computed a spatial and temporal average around each estimated (or interpolated) whale position. The spatial means were calculated in 0.25° bins (approx. 27 km^2^), and the time mean within the 15-day period prior to the whale location. The temporal average was set 15 days prior to whale location as an attempt to consider the time of development of intermediate trophic levels in planktonic communities. These spatial and temporal means allowed us to fill gaps in satellite data availability due to cloud coverage, as well as to remove any potential local noise. We averaged over 0.25° bins rather than over 0.1° bins because this did not qualitatively change our results, and using the 0.1° bins removed some locations from the analysis.

SST was extracted from a daily SST dataset produced and distributed by JPL (http://ourocean.jpl.nasa.gov/SST/, G1SST product) at a 1 km spatial resolution. For consistency with the surface chlorophyll-*a* analysis, we used a mean value within a 0.25° bin around each whale location.

For the open-ocean dataset, daily geostrophic currents were computed from the daily merged and gridded satellite altimetry product produced by Aviso (http://www.aviso.altimetry.fr/) with a spatial resolution of 0.25° (latitude, longitude).

Because altimetry-derived ocean velocity measurements are noisier closer to a coast, surface currents for the coastal dataset were not available from Aviso products. Instead, we used a climatological estimate of a geostrophic current from a regional ocean model configuration. The model configuration is based on the NEMO ocean general circulation modelling system [69] and is a subset from the global 1/12° resolution (i.e. cell size approx. 9 km) configuration described by [70] covering the southwestern Indian Ocean subdomain (31–66.25° E; 3–29° S). This model successfully reproduces the major currents (mean and variability) as well as water masses’ properties. For the purpose of this study, only winter (June to October) surface currents are considered and used to calculate the temporal mean and the standard deviation over years (1995–2009), which constitute the climatology from which the current data were extracted underneath the whale tracks.

### 2.5. Movement datasets

From the 19 whales that displayed coastal movements, 11 were females (six females with calves and five females without calves; electronic supplementary material, figures S2*a* and S2*b*) and eight were males. Whale movements were analysed using linear mixed-effects models (LMMs; see below for details of analysis) considering the logit of behavioural mode values and whale active swimming speed values as response variables, and SST, bathymetry, DIST, slope and current speed as explanatory variables.

Seven whales displayed oceanic movements including six females and one male. These oceanic movements were analysed using LMMs considering the logit of behavioural mode values, whale active swimming speed and whale deviation from the current values as response variables, and SST, bathymetry, slope, current speed and the surface CHL concentration as covariates.

### 2.6. Behavioural metrics

Two metrics were used to characterize the whale movements: (i) the behavioural mode from SSSM outputs described earlier (*see data processing*), and (ii) the whale active swimming speed. The observed whale speed vector (*T*) was estimated by computing the distances, in the *x* and *y* directions, between the two consecutive locations and then dividing by the time elapsed. It was systematically computed as

2.1 $$T(t)=\frac{\left[X(t+\Delta t)-X(t)\right]}{\Delta t},$$

where [*X*(*t*), *X*(*t* + Δ*t*)] are the two successive positions over a time interval, *t*. The observed whale speed vector (*T*) can also be written as the sum of the whale's active swimming vector (*A*) and the current vector (*C*) (e.g. [71])

2.2 T(t)=A(t)+C(t).

In other words, the mean active swimming speed (*A*) computed over the time interval [*t*, *t* + Δ*t*] can be expressed as the difference between *T*(*t*) and the averaged current speed during the same time interval (namely [*C*(*t*) + *C*(*t* + Δ*t*)]/2; e.g. Galli *et al.* [72]). To simplify the notation, *A*(*t*) will be denoted as *A*, *T*(*t*) as *T* and [*C*(*t*) + *C*(*t* + Δ1)]/2 as *C*; so equation (2.2) is rewritten as

2.3 A=T−C.

The resulting tracks from the SSSM are made of positions estimated with a fixed sampling period Δ*t*  =  12 h. To extract environmental variables under the whale tracks, we first linearly interpolated locations every 6 h between the positions estimated every 12 h by the SSSM (using R package adehabitat, function redisltraj; [73]). Then the computed whale active swimming speeds were assigned to each interpolated location and environmental variables were extracted for these locations. The latter positions were used in the modelling analysis to link the active swimming speed metric and environmental variables.

### 2.7. Statistical analysis

All statistical analyses were performed using R Program v. 3.1.2 (R Core Team 2014). We fitted a series of LMMs using the R software package nlme (function lme; [74]) following the steps described in Zuur *et al.* [75] to examine the relationship between three response variables (behavioural mode, active swimming speed, angle between whale's heading and surface current) and the explanatory environmental variables. Behavioural state is the proportional likelihood of exhibiting localized movements, ranging between 1 (transiting) and 2 (localized). As a consequence, behavioural mode values were logit-transformed before the analysis [76]. An autocorrelation term (corAR1) was added to account for the lack of temporal independence within telemetry data for each whale [75]. The individual whale was included as a random term. Both predicted location associated with missing environmental values and outliers (values that were ecologically unreasonable to include) were removed from analysis. Non-collinearity was verified between continuous variables using Pearson's correlation (coef ≤ 0.5) and the variance inflection factor (VIF) [75], and one of each highly correlated pair was removed. Explanatory variables were standardized (centred and scaled) to facilitate model convergence and enable comparison of their contribution (using their corresponding slope coefficients). Model selection was performed using likelihood ratio tests starting from a full model with terms retained only if they improved the fit (*p* < 0.05, [77]). The results were then evaluated such that the most parsimonious model was also the model with the lowest Akaike's Information Criterion (AIC). The resulting optimal model was then fitted using restricted maximum-likelihood (REML). The normality and homogeneity of residuals were checked graphically [77,78].

### 2.8. Oceanic movements: surface current and trajectory

We calculated angles between A and C vectors (A–C angles, angles between current and whale), named compensation angles, as a means to estimate how whales orient their swimming direction with respect to the current. Angles close to 0° indicate that whale movements were following current direction, whereas angles close to 180° indicate movements oriented against the surface current. We assessed the respective contribution of currents (*C*) and whale's swimming (*A*) on observed whale movements (*T*) by projecting, respectively, the *C*-vector onto the *T*-vector (PCT) and *A*-vector onto the *T*-vector (PAT) (see Galli *et al.* [72]) and electronic supplementary material, S1).

## 3. Results

### 3.1. General tracking information

**Figure 2.**
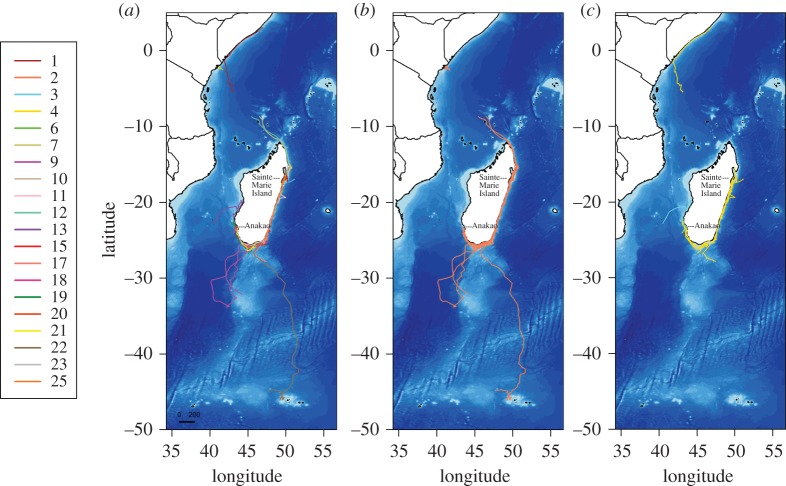
Trajectories of tracked humpback whales in the Madagascar breeding ground (*n* = 20) (*a*). Trips shorter than 8 days are not represented. Females are represented in orange (*b*), males in yellow (*c*) and an individual of unknown sex in blue (*c*).

Whales were tracked for an average duration of 24.2 days (range 2–58 days) yielding 5631 locations raw Argos location. Five tracks shorter than 8 days were discarded from our analysis, providing us with 20 trips, or 1039 estimated locations after application of the SSSM (11 females, eight males and one of unknown sex; table 1, figure 2 and electronic supplementary material, figure S4*a*,*b*).

Whales travelled along the east, southwest and southern coasts of Madagascar with a high inter-individual variability in swimming direction (figure 2 and electronic supplementary material, figure S2*a*,*b*). The distances travelled during the tag transmission period (calculated from estimated SSSM positions) ranged from 488 to 4575 km, with an average (±s.e.) travelling speed of 31 ± 1 km per day (table 1). While most tagged individuals stayed relatively close to the Madagascar coast, one displayed a southward migration and reached as far as the Crozet Islands (approx. 2000 km south of Madagascar coast; whale 22 and another one (whale 9) visited the Walters Shoals seamount before coming back to the south of Madagascar (figure 2); Cerchio *et al*. [62] discuss in greater detail the movements of tagged whales within Madagascar waters and destinations of whales departing from Madagascar.

All humpback whales in our study spent time in the coastal waters of Madagascar, with the vast majority of received locations (78%) classified as coastal (figure 1). Among those, 12 whales (2, 3, 6, 10, 11, 15, 18, 19, 20, 21, 23 and 25) remained exclusively over the continental shelf of Madagascar for the duration of tag transmission (electronic supplementary material, figure S2*a*,*b*). Seven whales (1, 7, 9, 12, 13, 17 and 22) stayed within coastal waters of Madagascar before heading toward the deep waters (electronic supplementary material, figure S2*a*,*b*). The mean tag transmission was slightly longer (Wilcoxon test, *p* < 0.001) for tracks containing an oceanic part (33 ± 7 days) than for tracks that were only coastal (26 ± 3 days). Most animals that undertook oceanic movements were females (five), with one male, and one was of unknown sex. Whales spent more time per unit area in coastal waters (4 ± 0.12 h per 0.1° × 0.1° bins) than in oceanic waters (2 ± 0.07 h per 0.1° × 0.1° bins).

### 3.2. Coastal movements

#### 3.2.1. General characteristics of movements and habitat in coastal areas according to sex and reproductive status

Overall, females used shallower waters (191 ± 20 m) than males (418 ± 48 m) on the shelf and in adjacent areas (electronic supplementary material, table S1 and figures S2*a* and S2*b*). Females spent a significantly higher portion of their time over the shelf (less than or equal to 200 m water depth) than males (69 ± 5%, and 45 ± 10%, respectively, permutation *t*-test, *p* = 0.034). However, there was no significant difference in the mean DIST between females (15.1 ± 2.8 km) and males (20.58 ± 3.9 km) (permutation *t*-test, *p* = 0.28) (electronic supplementary material, table S1 and figure S3). Similarly, there was no significant difference between females with calves and females without calves in the mean DIST (12.1 ± 3 km and 19 ± 4 km, respectively, permutation *t*-test, *p* = 0.2) or mean water depth (187 ± 58 m and 233 ± 66 m, respectively, permutation *t*-test, *p* = 0.5). Mean SST in the area used by whales ranged between 21 and 26°C (24 ± 0.04°C). The mean current speed in the same area was 0.29 ± 0.01 m s^−1^.

Transiting behaviour (consistent/directional movement, behavioural mode approaching 1; *see data processing*) was found in higher proportion in females (58%) than males (38%) (electronic supplementary material, figures S4–S6). Likewise, in coastal habitat, the mean behavioural mode of females was significantly lower than the mean behavioural mode of males (1.25 ± 0.12 and 1.52 ± 0.39, respectively, permutation *t*-test, *p* = 0.016), suggesting that males performed more localized movements (variable/non-directional movement, behavioural mode approaching 2; *see data processing*) in coastal areas than females (electronic supplementary material, figure S4*a*,*b*). There was no significant difference between the behavioural mode of females with a calf (1.30; 6 individuals), and females without a calf (1.1; 5 individuals) (permutation *t*-test, *p* = 0.26). The females' observed speed was not significantly lower than males' (0.85 ± 0.11 m s^−1^, 0.88 ± 0.11 m s^−1^, respectively; permutation *t*-test, *p* = 0.8). Additionally, the observed speed of females with a calf (0.82 ± 0.08 m s^−1^) was not significantly different from females without a calf (0.89 ± 0.13 m s^−1^) (permutation *t*-test, *p* = 0.69).

#### 3.2.2. Influence of environmental conditions on both female and male behavioural mode and swimming speed

The influence of environmental variables on the behavioural mode of females was most parsimoniously described by a model including water depth and distance to shore (table 2; electronic supplementary material, table S2). Namely, the behavioural mode (i.e. probability of performing localized movements) was positively correlated to depth and to DIST. By contrast, the most parsimonious model describing the behavioural mode of males included all environmental variables except DIST (collinear with the depth) but none was associated significantly with movement patterns (table 2).

**Table 2.** Summary of regression coefficients from the most parsimonious models (LMMs) relating b-mode (logit) or whale swimming speed to environmental parameters during coastal movements (for the females and males) and oceanic movements. Coefficients are presented ± s.e. with their *p*-value associated. Significant parameters are highlighted in italicized characters. Parameters not included in the full model are indicated by a black box, and those included in the full models but not retained in the model selection by an em-dash.

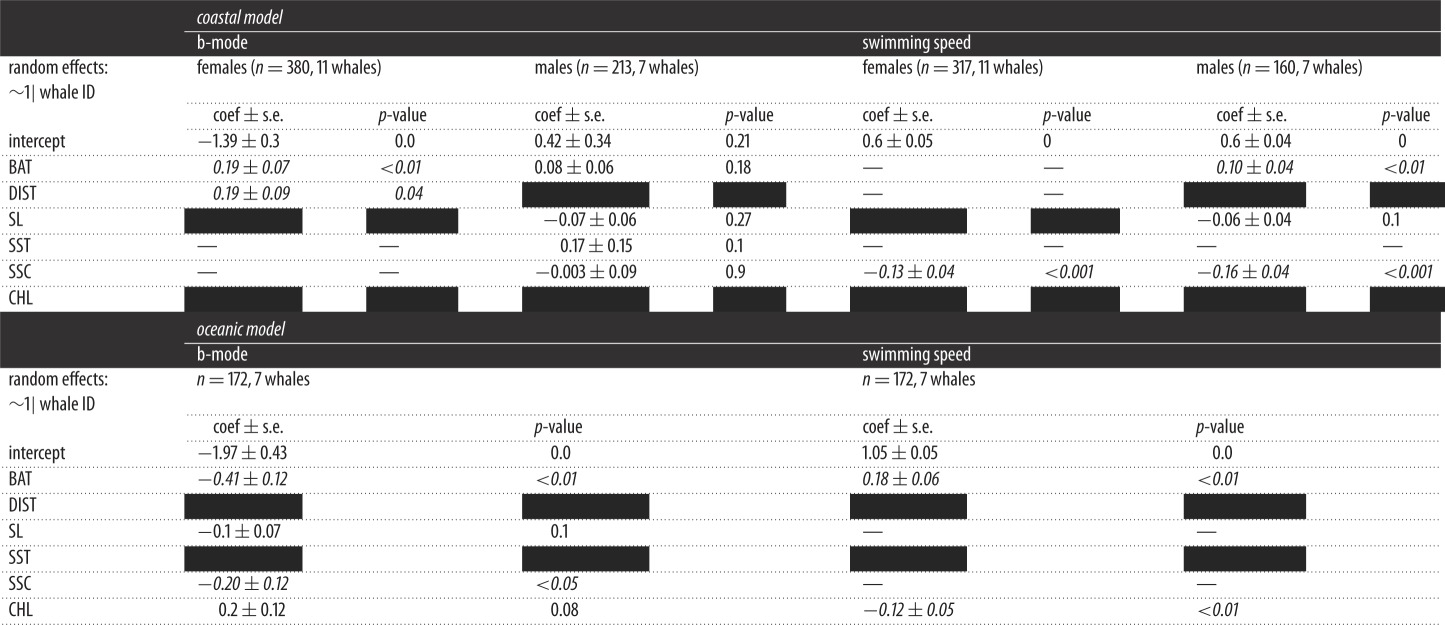

The active swimming speed of females was most parsimoniously described by a model including only the speed of the surface current (table 2; electronic supplementary material, table S2): female active swimming speed increased with decreasing current speed. The most parsimonious model describing the behavioural mode of males included all environmental variables except DIST (collinear with the depth; table 2). The model indicated swimming speed was positively correlated with water depth and negatively correlated with current intensity.

### 3.3. Oceanic movements

#### 3.3.1. General characteristics of movements in oceanic areas

**Figure 3.**
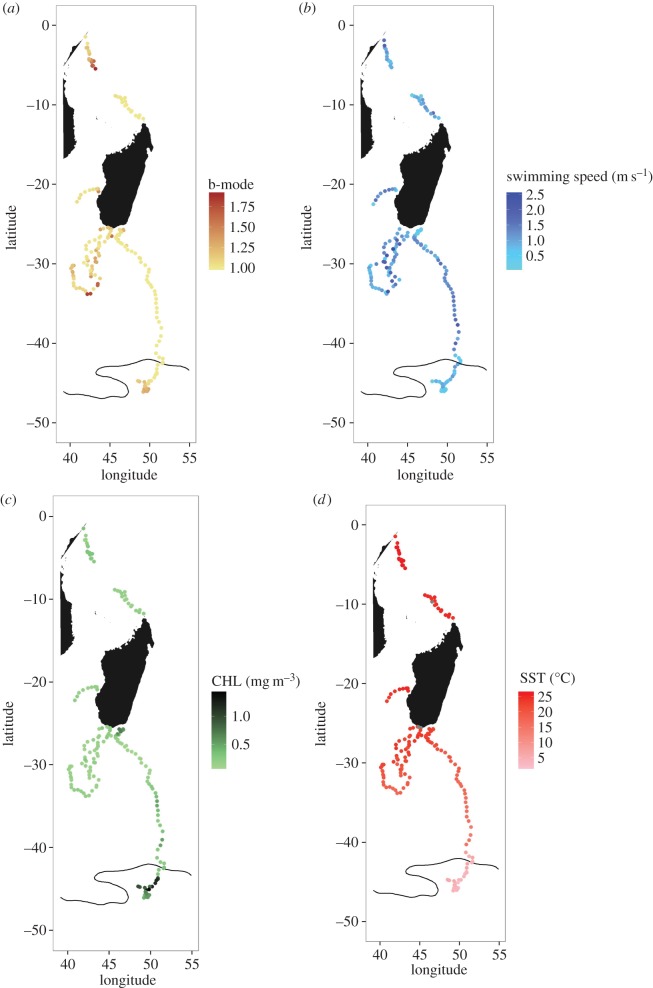
Locations for whale oceanic movements. Values of the b-mode (*a*), the active swimming speed (*b*), the CHL (*c*) and the SST (*d*) are expressed for each location. Polar Frontal Zone from Roquet *et al.* [79] is represented by the black line.

During oceanic movements, humpback whales travelled over deep waters (mean depth 2944 ± 105 m), and encountered an average current speed of 0.3 ± 0.02 m s^−1^ (electronic supplementary material; table S3). The CHL along their tracks ranged between 0.08 and 1.4 mg m^−3^ (mean of 0.3 ± 0.02 mg m^−3^). The mean SST values ranged from 2 to 26°C (mean of 18 ± 0.6°C). The highest mean CHL concentration (0.5 ± 0.04 mg m^−3^, max: 1.4 mg m^−3^) and the lowest mean SST (10.8 ± 0.9°C, min: 2°C) were encountered by whale 22, where it arrived at the Polar Frontal Zone (approx. 45° S) in late September (electronic supplementary material, figure S2*b*; figure 3).

The behavioural mode associated with oceanic movements of whales ranged from 1 to 1.9 (1.14 ± 0.01). Transiting behaviour (consistent/directional movement, defined here as a behavioural mode less than 1.25) was found during 79% of the oceanic movements (figure 3). Observed speed was significantly higher in deep waters (migration track not included) than in coastal waters (1.15 ± 0.08 m s^−1^ and 0.86 ± 0.08 m s^−1^, respectively, permutation *t*-test, *p* = 0.03).

#### 3.3.2. Influence of environmental conditions on behavioural mode and swimming speed

The behavioural mode of oceanic movements was most parsimoniously described by a model including only two environmental variables: current speed and water depth; no significant influence of slope or CHL concentration was detected (table 2; electronic supplementary material, table S2). The behavioural mode (i.e. transiting behaviour, approaching 1, and localized behaviour, approaching 2) was negatively related to both water depth and current speed.

Active swimming speed was most parsimoniously described by a model including only water depth and CHL concentration (table 2; electronic supplementary material, table S2). Whales swam faster in deeper waters and in areas with lower CHL concentration (figure 3). No significant effect of slope and current speed was detected.

It is noteworthy that for all oceanic movements, the mean active swimming speed was always found to be twofold to fivefold greater than the mean surface current speed they encountered.

#### 3.3.3. Ocean currents’ influence on animal direction in oceanic habitat

**Figure 4.**
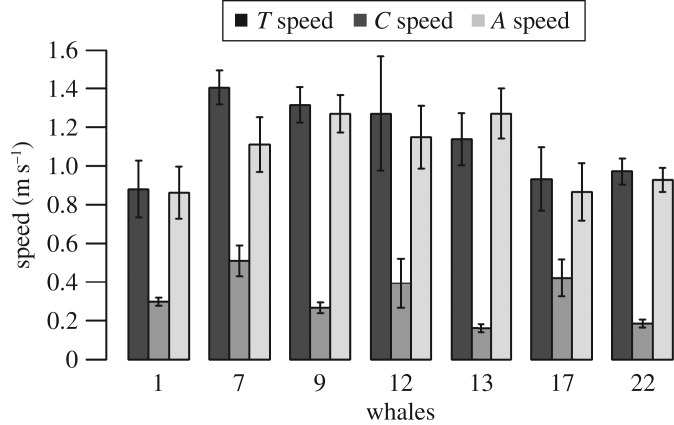
Mean speed ± s.e. of the observed whale speed (*T*), the current (*C*) and whale's active swimming speed (*A*) vectors for each individual whale during oceanic movement. The value of *C* speed shows the mean intensities of the currents along each whale trajectory, while *A* provides a measure of each whale's active swimming effort.

**Figure 5.**
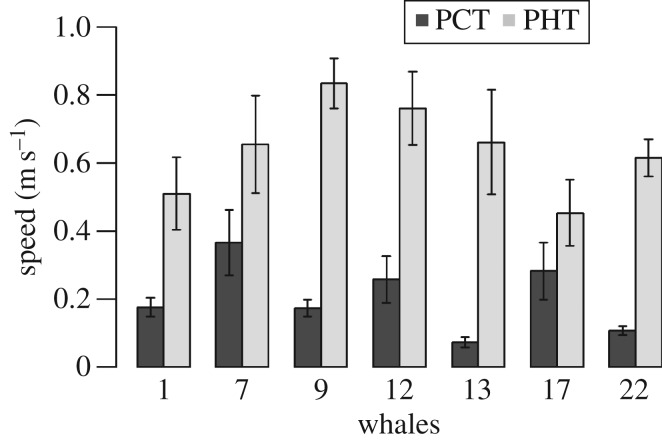
Mean ± s.e. projections of the *C* vector on the *T* vector (PCT) and the *A* vector on the *T* vector (PAT) for each whale.

The mean magnitude of *C*, the surface current, along oceanic whale tracks ranged from 0.16 to over 0.51 m s^−1^, while the mean magnitude of *A*, the active swimming vector, ranged from 0.87 to over 1.27 m s^−1^ and was larger than *C* for all whales (figure 4). Only whale 13, which moved offshore towards the Mozambique Channel showed an *A* higher (1.27 m s^−1^) than *T* (0.97 m s^−1^). In addition, in all pelagic whale movements, the PATs (the projection of the active swimming vector onto the observed direction vector) were higher than the PCT (the projection of the current vector onto the observed direction vector (figure 5). This demonstrates that the whales actively swam regardless of current speed.

**Figure 6.**
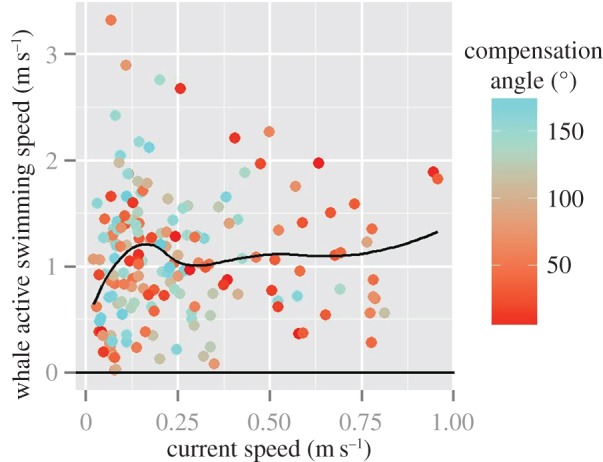
Whale active swimming speed (*A*) by current speed (*C*). Colour scale shows the compensation angles.

**Figure 7.**
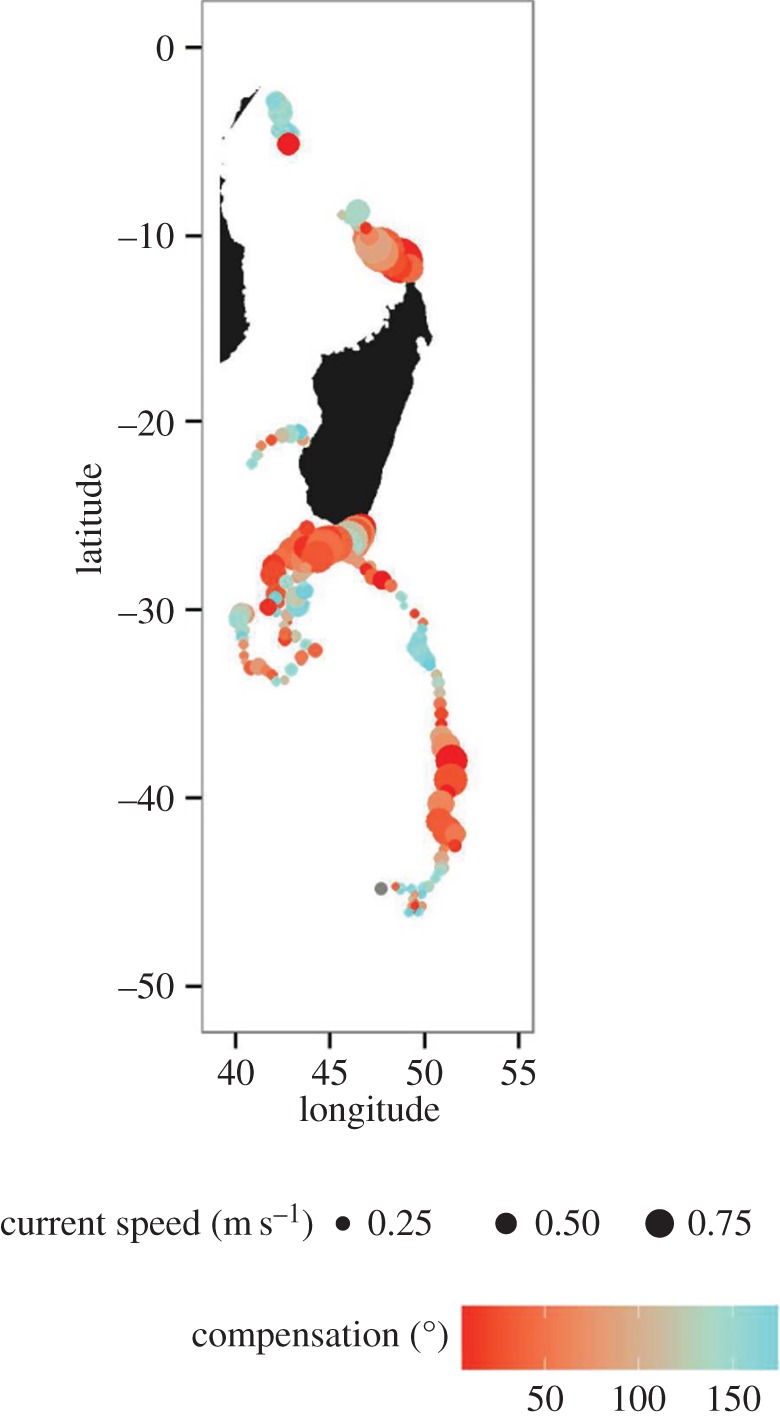
Current speed for each whale oceanic location. Colour scale shows the compensation angles.

As shown in figure 6, even though compensation angle and current speed were negatively correlated (Pearson's *r* = −0.27, *p* < 0.001; d.f. = 173), the whale active swimming speed did not depend on current speed, and plateaued at around 1 m s^−1^ for current speeds increasing from 0.2 to 1 m s^−1^. As further shown in figure 7, when whales moved away from Madagascar to the north or the south, the compensation angle was low and then varied according to each individual whale's path (electronic supplementary material, figures S7*a* and 7*b*). Whales were oriented in the same direction as prevailing currents when the currents were strong in the oceanic sectors (figure 7). We note, however, that no environmental parameter was retained in the deviation model with compensation angle and current speed.

## 4. Discussion

In this study, we used satellite tracking of 25 humpback whales to investigate the links between breeding movements and habitat use of humpback whales according to sex and breeding status in Madagascar breeding grounds. By modelling movements of tagged humpback whales against a suite of environmental parameters, we were able to make inferences on habitat preferences between males and females during the breeding season in Madagascar. Our results showed no significant habitat differences (i.e. BAT, DIST) between females without calves and females with calves, possibly due to a low resolution from available data. However, our models suggested that female breeding habitat extends beyond the shallow coastal waters and males tend to not be very much influenced by oceanographic factors in their breeding habitat, at least for the parameters that we examined at this spatial scale. In oceanic habitats, the active swimming speed accounts for a large proportion of the whale's observed speed, while the observed direction of tagged whales tends to align to the current direction when the current intensity was high.

### 4.1. General patterns of coastal and oceanic movements

Humpback whales preferentially used the continental shelf, which is consistent with what has been observed in many other breeding areas. In addition, they spent, on average, more time per unit area when engaged in coastal movements than during oceanic movements. This concurs with the lower observed whale speed and more localized movements (i.e. variable/non-directional movement, lower behaviour mode) found in coastal waters. Altogether, these results suggest that the shelf is a key habitat on which whales exhibited a large amount of localized movements, most likely related to breeding activities (e.g. searching, pairing, mating and resting; [80]). Both females and males increased their active swimming speed with decreased current speed, suggesting that in coastal habitats when whales were engaged in movements that could correspond to mate-searching movements, current speed did not influence their movement patterns [81]. By contrast, in oceanic habitats currents influenced whale behavioural mode and could impact the whale headings. Our models did not show any influence of SST on humpback whale movement patterns, perhaps as a result of the low variations in SST within the Madagascar coastal region, and/or because SST does not impact the way whales use their habitat once they reach the breeding grounds.

### 4.2. Coastal movement of females and males in relation to habitat characteristics

While all whales spent more time visiting coastal than oceanic areas, females performed more transiting than localized movements during their time in the coastal environment, as indicated by their mean behavioural mode [62]. Although no significant differences were found in behaviours and habitat use between females with calves and females without calves, females with calves tended to perform more localized movements and occurred in nearer-shore areas than females without calves. The most important environmental parameters affecting the movement patterns of females were the water depth and the distance from shore. Within coastal habitat, localized movements of females were performed at greater distances from the shoreline and in deeper waters, suggesting that they used the offshore habitat more intensively than expected. Because of the difficulty of identifying females in the field in the absence of a calf, little information exists on non-parous females' movements and the potential influence of habitat features. Non-parous females (migrating to the breeding grounds solely to mate) can be found in competitive groups or pairs that are mostly observed in deeper waters, farther from shore [28,29,82]. This is consistent with our results, suggesting that non-parous females preferentially use areas favourable for reproductive behaviours, i.e. the deeper offshore coastal waters where competitive groups and possibly singing males are likely to be found. Previous studies show that mother–calf pairs have a preference for shallow coastal waters and areas close to shore [16,28,29,83,84]. Although many previous research studies including boat-based and shore-based studies of humpback whales spent little time in offshore areas, other studies sampled both inshore and offshore areas in a relatively comparable way and found mother–calf pairs preferentially in coastal waters (e.g. [67,72,74,75,85,86]). It may be that females with calves change movement patterns and habitat use across the season as the calf matures by moving more extensively into offshore areas and deeper waters on the shelf. It could be expected that females introduce breeding locations to their newborns although this has never been demonstrated [29,87]. Our tracking data revealed that females, including mothers with calves, use offshore areas on the shelf, suggesting that females probably exploit a more extended range of reproductive habitat than previously thought.

Our models did not reveal any significant effect of environmental parameters on the type of movements (localized or transiting) performed by males. However, male active swimming speed was positively influenced by depth, indicating that animals slowed down in shallow waters. The absence of influence of key environmental parameters on the behavioural mode suggests that males were more influenced by social factors such as female occurrence, possibly in order to increase the number of opportunities to interact with receptive females. The mating system of humpbacks whales includes alternative mating strategies adopted by males (e.g. engaging direct competition with other males, singing or escorting) presumably to successfully obtain mating with reproductive females [8,88]. Male songs are known to play a role in the humpback whale mating system [8,89], suggesting that acoustic conditions could influence the occurrence and habitat utilization of singing males [82,90]. For example, in Puerto Rico, singers tend to be found on ledges, close to the shelf, which may be favourable geographical features to spread out the song or detect songs of other males [5]. However, several studies have suggested that male movement patterns may be mostly driven by mating prospects, and by the temporal distribution of receptive females, rather than by favourable singing habitats [13,82,91–93].

### 4.3. Oceanic movements in relation to habitat characteristics

Overall, in oceanic habitat, whales displayed highly directional movements in deep waters between breeding sites but also performed more erratic movements in oceanic shallow habitats such as Walters Shoals seamount or the Crozet Plateau during migration.

#### 4.3.1. Directional movements between breeding sites

The tracks of tagged whales revealed that whales used oceanic habitats during the breeding season and performed highly directional and consistent movements (i.e. transiting behaviours) both in deep open waters and in areas with strong currents. Non-migrating animals travelled faster in oceanic than in coastal habitats with an average observed speed of 1.21 ± 0.08 m s^−1^. This value is slightly lower than the speed found in whales migrating southwards from Moheli and Mayotte in the Comoros Archipelago [94], higher than whales from the eastern South Atlantic [95], but is comparable to swimming speeds of humpback whales migrating between breeding and feeding grounds in the South Atlantic (Brazil; [57]), the North Atlantic (Caribbean Sea; [26]) and in the North Pacific Ocean (Hawaii; [96], Mexico; [97]). This study was the first to assess humpback whale swimming speed between different breeding sites within a breeding region. Our findings suggest that during offshore movements between breeding sites, humpback whales generally swam at a rate of travel similar to the mean travelling speeds of migrating whales.

#### 4.3.2. Movement on Walters Shoals seamount

One individual (whale 9) displayed localized movement patterns and moved slowly over the offshore Walters Shoals seamount. Our models indicate that humpback whales exhibited localized movements and a slower active swimming speed in response to shallow sea floor depth, chlorophyll concentration and low current speed. Walters Shoals seamount may constitute either previously undescribed breeding habitat or potential opportunistic feeding habitat. Seamounts are shallow geographical features frequently found at low latitudes, suggesting that they represent important habitats for humpback whales during both the breeding and migratory periods [20]. Humpback whales have been previously observed in the Walters Shoals seamount in September, the same period as whale 9 (M. Le Corre, 28 October 2014, personal communication). Furthermore, occurrence of humpback whales over shallow seamounts during the breeding period has been documented elsewhere in the southwestern Indian Ocean (La Perouse seamount off Reunion Island; [98]) and in the Pacific Ocean (Antigonia seamounts; [19]). While relatively low instantaneous chlorophyll concentrations (ranging from 0.1 to 0.2 mg m^−3^) were associated with the whale positions on the Walters Shoals seamount, the monthly mean chlorophyll concentrations were higher (ranging from 0.5 to 1 mg m^−3^; averaged for September 2013) than adjacent waters located further north (electronic supplementary material, figure S8). The Walters Shoals are located downstream of productive regions: south of Madagascar and the subtropical South Indian Ocean countercurrent [53,99,100]. A number of humpback whales have also been sighted on the Walters Shoals seamount in summer (November and December), a time of increased productivity in the area [31,50,101]. The shallow waters of seamount habitats are favourable for humpback whale breeding activities, yet the abrupt topographies and the occurrence of localized physical processes (e.g. tides, eddies and upwelling) surrounding seamounts are known to favour prey aggregation attracting marine mammals [102–104] such as humpback whales [56,105]. Consistently, the Walters Shoals area is also known to be a seabird foraging hot spot [106,107]. Further investigation and larger sample sizes are needed to fully understand the presence of humpback whales in that region at that time of the year.

#### 4.3.3. Migration

Our results indicate that multiple whales engaged in oceanic movements decreased their swimming speed in response to high chlorophyll concentrations. One individual (whale 22) migrated south and spent 6 days in productive waters (i.e. higher chlorophyll concentration) in the west part of the Crozet Plateau before the tag stopped transmitting. This suggests that this individual very likely stopped to feed in this area where a phytoplankton bloom is present all year long [108] and supports zooplanktonic populations such as copepods [109]. Fossette *et al*. [94] reported a humpback whale migration from Mayotte towards northwest of the Crozet Plateau, and also suggested that humpback whales could forage in that area either before migration to Antarctic feeding grounds, or could remain within the plateau area during summer [94]. Humpback whales are also regularly observed from fishing vessels operating on the Crozet Island Plateau in spring and early summer [110]. Our individual movement data support that humpback whale stop over on the Crozet Plateau probably to feed, at least en route to high latitude foraging areas.

#### 4.3.4. Movement direction

We investigated the influence of currents on the whale active swimming speed and direction of travel during the offshore sections of tagged whale non-migratory movements and one migratory movement. We note that, to our knowledge, this is the first time current influence on whale movements has been investigated using current values extracted under the whale positions. Humpback whales exhibited highly directional travelling movements over deep waters. Our results showed that active swimming speed stayed around 1 m s^−1^ across the range of current speed encountered by the whales (up to 1 m s^−1^) close to the optimal velocity of 1.1 m s^−1^ found by Braithwaite *et al*. [111]. By contrast, the direction of tagged whales tended to be closer to the current direction when the current intensity was high, such as at the northern and southern tip of Madagascar. We found that whales tended to follow the prevailing currents when they moved away from Madagascar, thereby potentially using a strategy for minimizing their energy expenditure. Then, depending on their headings, they may adjust their movement direction to the local current conditions. Few studies have investigated the influence of ocean currents on swimming speed and direction of large migratory species, and previous work has mostly concerned leatherback turtle (*Dermochelys coriacea*, [70,71,111–114]). A tracking study of humpback whales migrating from the Brazilian breeding grounds to the South Atlantic feeding grounds has suggested that whales would tend to keep a constant heading regardless of current direction, but this analysis was based on mean currents at the regional scale [115]. Chapman *et al.* [81] discussed the same results from a theoretical point of view and suggested that whales could use a compensation strategy that involves an animal altering its heading into the flow to achieve a track coincident with its desired direction, regardless of current direction. However, these authors also noted that animals could not persistently compensate for currents over very long journeys, and might adjust their strategy to the local ocean circulation context, thereby alternating phases of active downstream strategy (heading alignment along current direction) and compensation strategy. In our study based on real-time, local current data extracted from underneath the whale oceanic tracks, we found that whales did not compensate for current direction in the strongest current area. This suggests that whales aligned their headings with the local current directions to exploit strong favourably directed ocean surface currents, and maximized their displacement speed and travelled distance in a given time. Thus, in non-migratory offshore movements (e.g. travel between breeding sites, short-term offshore travel), humpback whales could stop compensating at some point along the pathway by taking up their headings along with the current such as in the presence of strong currents.

## 5. Conclusion

Our satellite tracking-based study described movement patterns and habitat utilization of humpback whales on the Madagascar breeding grounds. During the winter breeding season, humpback whale habitat was not restricted to coastal waters, but also included offshore habitat. Whales from Madagascar used offshore habitats and moved to other coastal areas in the Indian Ocean (e.g. east coast of Africa). Humpback whale movement patterns were significantly related to bathymetric features, in both coastal and oceanic habitats. In the former, females spent most of their time on the shelf where they performed localized movements while at a distance from inshore areas. Our study showed that females (with or without a calf) use a wider range of breeding habitats than near-shore waters only. Unfortunately, our sample size was too small to investigate in detail the influence of environmental parameters on movements according to female subclasses. We also found that whales performed offshore movements during the breeding season and that their observed swim speeds were represented in large part by their active swimming speeds. While only seven whales performed offshore movements, we found that humpback whales do not constantly employ a compensation strategy but can alter their direction based on local currents. This study highlights the need to increase the sample size of tagged whales and extend the duration of the tag anchoring system to better identify different strategies of females (with or without a calf) within breeding seasons and to better assess the different winter movements. Our results improve our understanding on humpback whale movement patterns and habitat use in critical breeding coastal regions around Madagascar and adjacent oceanic habitat, which need to be taken into account to implement effective management and conservation strategies

## Supplementary Material

Supplementary material is available in a pdf file. Figure S1. Mean surface current (m/s) in the Southwest Indian Ocean during the winter 1995-2009 according to the SWIO12 climatological model. Southward branch of the South equatorial current represents the East Madagascar Current (EMC). Stars represent the well-known breeding areas of humpback whales in Madagascar (Cerchio et al. 2008, 2009). Figure S2a. Movements of each humpback whale tracked in this study (2012-2014) after application of SSSM to filtered Argos locations to estimate improved locations. The top left map shows the whale tracks removed from analyses. Note that two whale tracks are shown on each figure. The colour scale indicates the bathymetric depth (m). The temporal progression is represented with continuous colour gradients (the first initial location after tagging is represented in purple or brown and the final location is represented in pink or yellow). Stars and dotted lines indicate tracks with an oceanic part. Figure S2b. Movements of each humpback whale tracked in this study (2012-2014) after application of SSSM to filtered Argos locations to estimate improved locations. Note that two whale tracks are shown on each figure. The colour scale indicates the bathymetric depth (m). The temporal progression is represented with continuous colour gradients (the first initial location after tagging is represented in purple or brown and the final location is represented in pink or yellow). Stars and dotted lines indicate tracks with an oceanic part. Supplement S1. Projection of C on T vector (PCT) was calculated as C x cos (Ac-At) where C is the current speed, Ac is the direction of the C vector and At is the direction of the T vector. The projection of H vector on T one (PHT) is calculated in the same way with the appropriate changes. Table S1. Summary of environmental variables for each whale coastal track. Values are presented as mean ± se. Individuals include females (F) alone or with calves and males (M). Distance from shore is the average of distances between closest positive bathymetric value and each whale position. Stars indicate that mean values were computed on all location values whereas mean values used in statistical tests were computed by individual. Table S2.AIC values for top of linear mixed effects models describing the influence of environmental parameters on each behavioral metric (df = degrees of freedom, AIC = Akaike Information Criterion). Table S3. Summary of environmental variables for each whale oceanic movements. Values are presented as mean ± se. Figure S3. Density distributions of the distance from shore for females and males during coastal movements. Figure S4a. Maps of female coastal movements showing b-mode values for each estimated location. It ranges from 1, meaning low probability of localized movement and 2 meaning high probability of localized movement. In alphabetical order: whale 4, whale 7, whale 9, whale 10, whale 12, and whale 15. Figure S4b. Maps of female coastal movements showing b-mode values for each estimated location. It ranges from 1, meaning low probability of localized movement and 2 meaning high probability of localized movement. In alphabetical order: whale 20, whale 21 whale 22, whale 23, and whale 25. Figure S5a. Male coastal movements showing b-mode values for each estimated location. It ranges from 1, meaning low probability of localized movement and 2 meaning high probability of localized movement. In alphabetical order: whale 1, whale 2, whale 3, whale 6, whale 11, and whale 17. Figure S5b. Male coastal movements showing b-mode values for each estimated location. It ranges from 1, meaning low probability of localized movement and 2 meaning high probability of localized movement. In alphabetical order: whale 18, and whale 19. Figure S6. Density distributions of b-mode values for females and males during coastal movements. Figure S7a. Current speed for each whale oceanic location of whales 1, 7, 9 and 12. Colour scale shows the compensation angles. Figure 7b. Current speed for each whale oceanic location of whales 13, 17 and 22. Colour scale shows the compensation angles. Figure 8. Chlorophyll-a concentration (mg/m-3) in September 2013 on Walters shoal seamount obtained from the NASA AQUA MODIS satellite data (http://disc.sci.gsfc.nasa.gov/giovanni)

## Data accessibility

Our data are available at the Dryad Digital Repository: http://dx.doi.org/10.5061/dryad.73h36/1 [116].
