# Supplementary material for: Influence of environmental parameters on movements and habitat utilization of humpback whales (Megaptera novaeangliae) in the Madagascar breeding ground
Source: R Soc Open Sci. 2016 Dec 21;3(12):160616. doi: 10.1098/rsos.160616 (PMC5210686; doi:10.1098/rsos.160616)
Supplement: Supplementary material is available in a pdf file. Figure S1. Mean surface current (m/s) in the Southwest Indian Ocean during the winter 1995-2009 according to the SWIO12 climatological model. Southward branch of the South equatorial current represents the East Madagascar Current (EMC). Stars repres [file rsos160616supp1.pdf]

Influence of environmental parameters on movements and habitat utilization of humpback whales (*Megaptera novaeangliae*) in Madagascar breeding ground.

Laurène Trudelle[1,2,3]*, Salvatore Cerchio[4,5], Alexandre N. Zerbini[6,7,8], Ygor Geyer[8], François-Xavier Mayer[9], Jean-Luc Jung[10], Maxime R. Hervé[11,12], Stephane Pous[2,13], Jean-Baptiste Sallée[2,] Howard C. Rosenbaum[14,] Olivier Adam[1,15], and Jean-Benoit Charrassin[2].

Supplement.

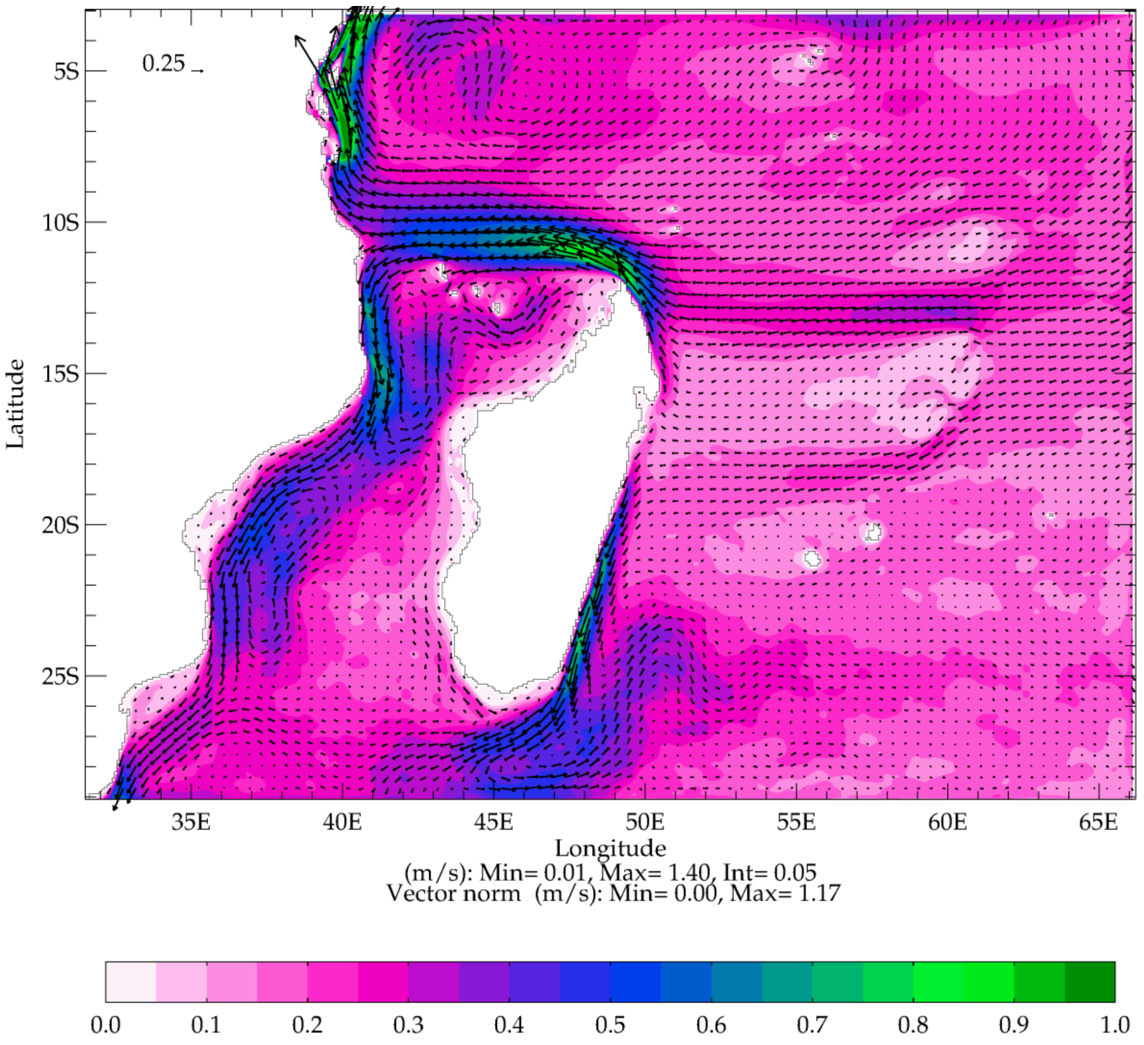

Figure S1. Mean surface current (m/s) in the Southwest Indian Ocean during the winter 1995-2009 according to the SWIO12 climatological model. Southward branch of the South equatorial current represents the East Madagascar Current (EMC). Stars represent the well-known breeding areas of humpback whales in Madagascar (Cerchio et al. 2008, 2009).

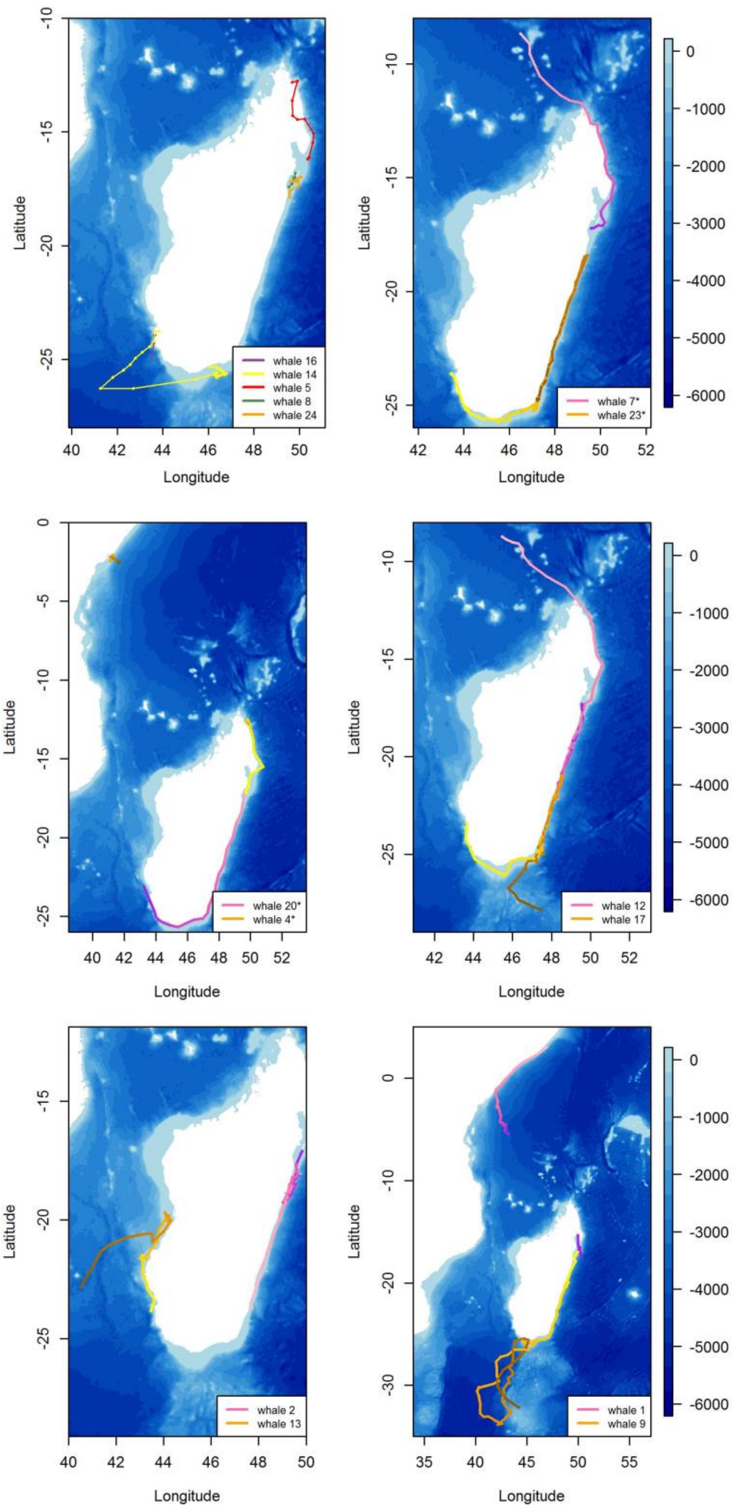

Figure S2a. Movements of each humpback whale tracked in this study (2012-2014) after application of SSSM to filtered Argos locations to estimate improved locations. The top left map shows the whale tracks removed from analyses. Note that two whale tracks are shown on each figure. The colour scale indicates the bathymetric depth (m). The temporal progression is represented with continuous colour gradients (the first initial location after tagging is represented in purple or brown and the final location is represented in pink or yellow). Stars indicate females with a calf.

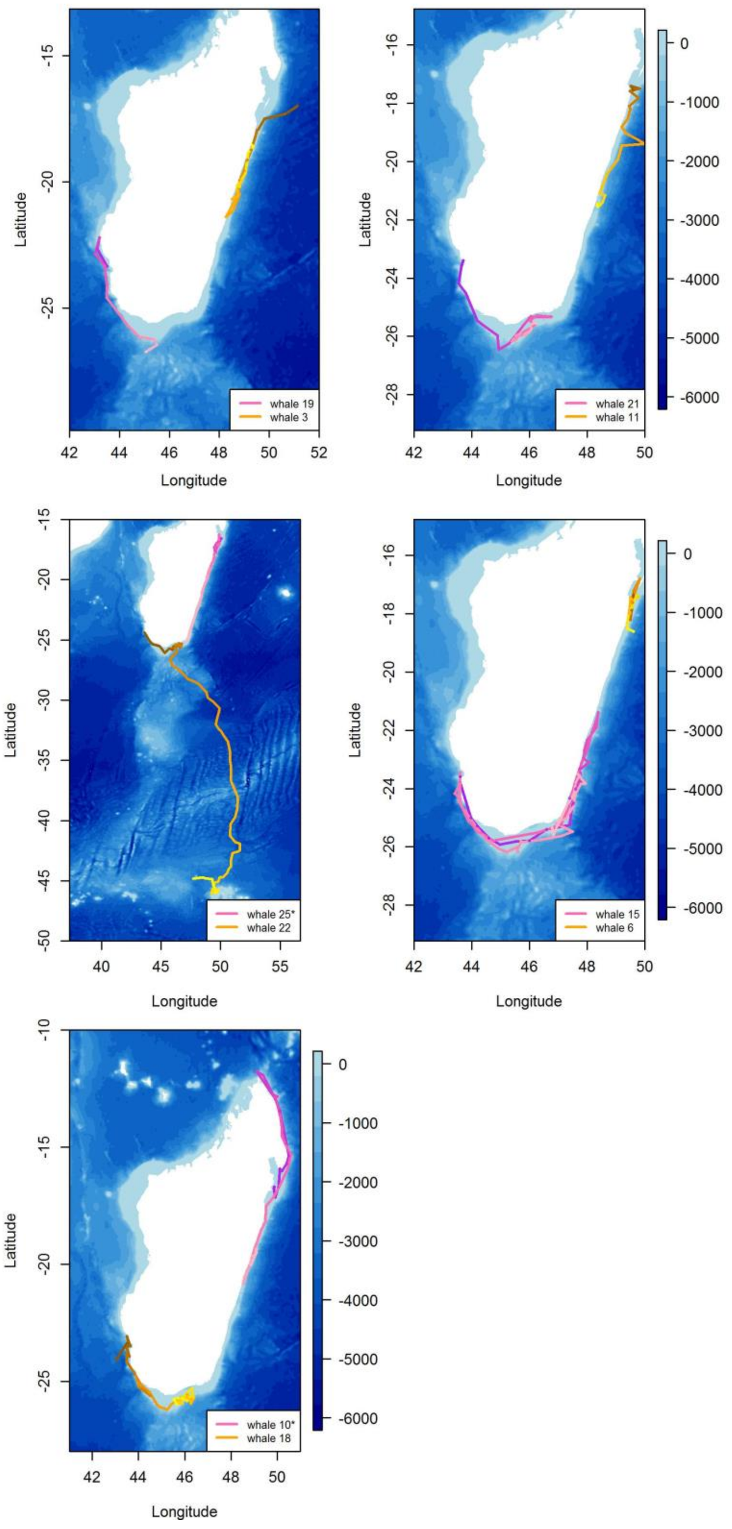

Figure S2b. Movements of each humpback whale tracked in this study (2012-2014) after application of SSSM to filtered Argos locations to estimate improved locations. Note that two whale tracks are shown on each figure. The colour scale indicates the bathymetric depth (m). The temporal progression is represented with continuous colour gradients (the first initial location after tagging is represented in purple or brown and the final location is represented in pink or yellow). Stars indicate females with a calf.

Supplement S1.

Projection of C on T vector (PCT) was calculated as C x cos (Ac-At) where C is the current speed, Ac is the direction of the C vector and At is the direction of the T vector. The projection of H vector on T one (PHT) is calculated in the same way with the appropriate changes.

Table S1. Summary of environmental variables for each whale coastal track. Values are presented as mean ± se. Individuals include females (F) alone or with calves and males (M). Distance from shore is the average of distances between closest positive bathymetric value and each whale position. Stars indicate that mean values were computed on all location values whereas mean values used in statistical tests were computed by individual.

Whale id	Sex	Group type	Bathymetry (m)	Slope (°)	Distance from shore (km)	Current speed (m.s)	Sea surface temperature (°C)
1.2	M	S	285 ± 105	2 ± 0.7	11.9 ± 2.5	-	25 ± 0.2
2	M	CG	178 ± 44	2 ± 0.4	10 ± 0.8	0.37 ± 0.01	24 ± 0.1
3	M	P	630 ± 222	3 ± 0.6	19 ± 5	0.4 ± 0.03	24 ± 0.1
4.1	F (with calf)	MC	450 ± 220	2 ± 0.5	10 ± 2	0.1 ± 0.02	24 ± 0.2
6	M	P	139 ± 110	0 ± 0.2	13 ± 2	0.22 ± 0.02	25 ± 0.1
7	F (with calf)	MC	213 ± 106	2 ± 1.1	12 ± 2	0.15 ± 0.02	25 ± 0.2
9	F	CG	212 ± 65	1 ± 0.3	19 ± 2	0.35 ± 0.03	24 ± 0.1
10	F (with calf)	MC	163 ± 48	1 ± 0.3	7 ± 1	0.25 ± 0.02	24 ± 0.1
11	M	CG	667 ± 208	3 ± 0.6	18 ± 4	0.39 ± 0.04	25 ± 0.1
12	F	CG	222 ± 60	2 ± 0.6	11 ± 1	0.37 ± 0.03	25 ± 0.1
15	F	P	469 ±89	3 ± 0.3	22 ± 2	0.37 ± 0.02	23 ± 0.1
17	M	P	249 ± 68	2 ± 0.4	18 ± 2	0.45 ± 0.03	23 ± 0.1
18	M	CG	542 ± 110	3 ± 0.7	32 ± 3.1	0.17 ± 0.009	23 ± 0.1
19	M	CG	1262 ± 233	4 ± 1	43 ± 10	0.21 ± 0.03	23 ± 0.2
20	F (with calf)	MBE	129 ± 63	1 ± 2.5	7 ± 1	0.24 ± 0.03	24 ± 0.2
21	F	CG	200 ± 53	2 ± 0.6	35 ± 4	0.17 ± 0.02	22 ± 0.1
22	F	NCG	135 ± 38	1 ± 0.2	27 ± 2	0.13 ± 0.02	23 ± 0.1
23	F (with calf)	MC	60 ± 9	1 ± 0.1	8 ± 1	0.27 ± 0.01	23 ± 0.1
25	F (with calf)	MC	34 ± 4	0 ± 0	7 ± 0.5	0.1 ± 0.02	25 ± 0.1
**Total**			**273 ± 21***	**1.8 ± 0.1***	**16 ± 0.6***	**0.29 ± 0.007***	**24 ± 0.04***
**Females**			**191 ± 20***	**1.4 ± 0.1***	**14 ± 0.6***	**0.27 ± 0.008***	**24 ± 0.05***
**Males**			**418 ± 48***	**2.4 ± 0.2***	**19 ± 1.3***	**0.34 ± 0.01***	**24 ± 0.07***

Table S2. AIC values for top of linear mixed effects models describing the influence of environmental parameters on each behavioral metric (df = degrees of freedom, AIC = Akaike Information Criterion).

**Model**	*df*	**AIC**	**ΔAIC**
**Coastal model - B-mode**			
**Males**			
BAT + SL+ SSC	8	1071.1	0
BAT+ SST + SSC	7	1072.4	1.3
BAT + SL+ SST + SSC *(full model)*	7	1072.81	1.71
BAT + SL+ SST	7	1073.21	2.11
SL + SST+ SSC	7	1088.66	17.56
**Females**			
BAT + DIST	6	1068.1	0
BAT + DIST+ SSC	7	1069.2	1.1
BAT	5	1070.3	2.2
BAT + DIST+ SST + SSC *(full model)*	8	1071	2.9
DIST	5	1074	5.9
**Coastal model - Swimming speed**			
**Males**			
BAT + SL + SSC	7	209.93	0
BAT + SSC	6	210.05	0.12
BAT + SL + SST + SSC + *(full model)*	8	211.83	1.9
BAT	5	213.92	3.99
SSC	5	221.08	11.15
**Females**			
SSC	2	381.9	0
SST + SSC	6	383.14	2.3
DIST + SST + SSC	7	384.52	1.2
BAT + DIST + SST + SSC (*full model)*	8	386.31	0
SST	2	393	6
**Oceanic model - B-mode**			
BAT + SL + SSC + CHL *(full model)*	8	430.44	0
BAT + SSC + CHL	7	430.62	0.18
BAT + SSC	6	431.86	1.42
BAT	5	433.62	3.18
SSC	5	437.18	6.74
**Oceanic model - B-mode**			
BAT + CHL	6	286.17	0
BAT + SSC + CHL	7	287.95	1.78
BAT + SL + SSC + CHL *(full model)*	8	289.9	3.73
BAT	5	290.95	4.78
CHL	5	293.72	7.55

Table S3. Summary of environmental variables for each whale oceanic movements. Values are presented as mean ± se.

Whale id	Sex	Group type	Bathymetry (m)	Distance from shore (km)	Current speed (m.s)	Chlorophyll a concentration ($mg.m^{-3}$)	Sea surface temperature (°C)
1.2	F	CG	3356 ± 199	239 ± 26	0.3 ± 0.02	0.3 ± 0.01	25.8 ± 0 .1
7	F (with calf)	MC	3638 ± 275	66 ± 9	0.5 ± 0.1	0.2 ± 0.01	24.8 ± 0.1
9	F	CG	3539 ± 194	508 ± 37	0.3 ± 0.03	0.2 ± 0.005	21.2 ± 0.3
12	F	CG	3183 ± 503	56 ± 12	0.4 ± 0.1	0.2 ± 0.005	24.5 ± 0.2
13	-	NCG	2261 ± 286	85 ± 16	0.2 ± 0.03	0.2 ± 0.008	24.2 ± 0.3
17	M	P	1783 ± 139	155 ± 27	0.4 ± 0.09	0.3 ± 0.07	22.5 ± 0.7
22	F	NCG	2493 ± 165	379 ± 40	0.2 ± 0.02	0.5 ± 0.04	10.8 ± 0.9
			**2944 ± 105**	**2 ± 0.22**	**0.3 ± 0.01**	**0.3 ± 0.02**	**18 ± 0.6**

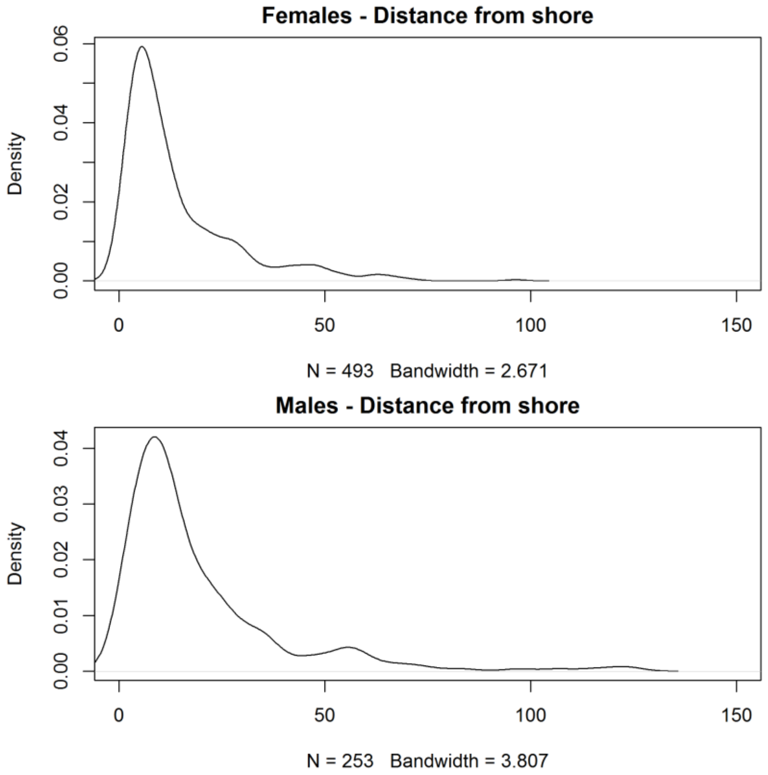

Figure S3. Density distributions of the distance from shore for females and males during coastal movements.

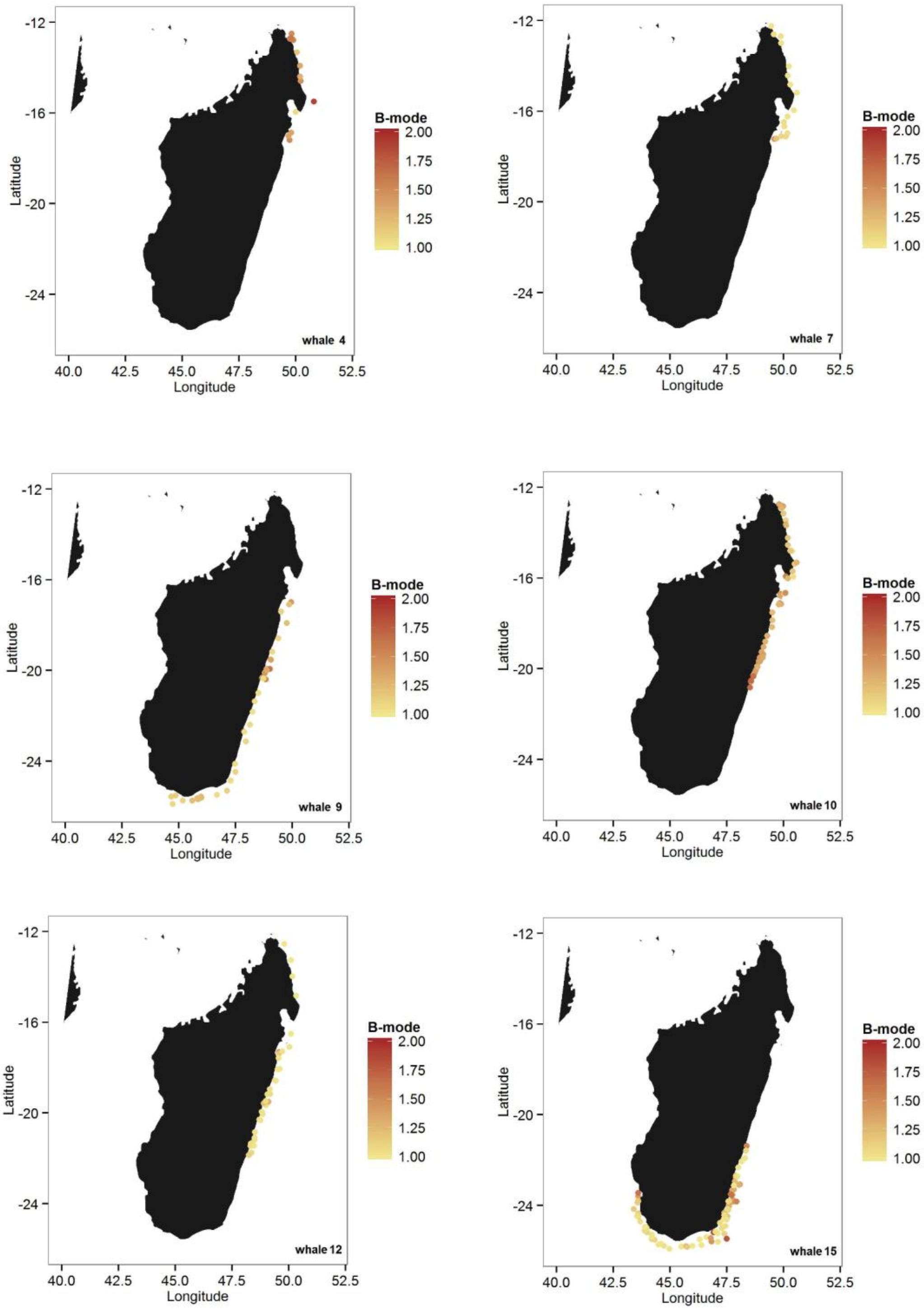

Figure S4a. Maps of female coastal movements showing b-mode values for each estimated location. It ranges from 1, meaning low probability of localized movement and 2 meaning high probability of localized movement. In alphabetical order: whale 4, whale 7, whale 9, whale 10, whale 12, and whale 15.

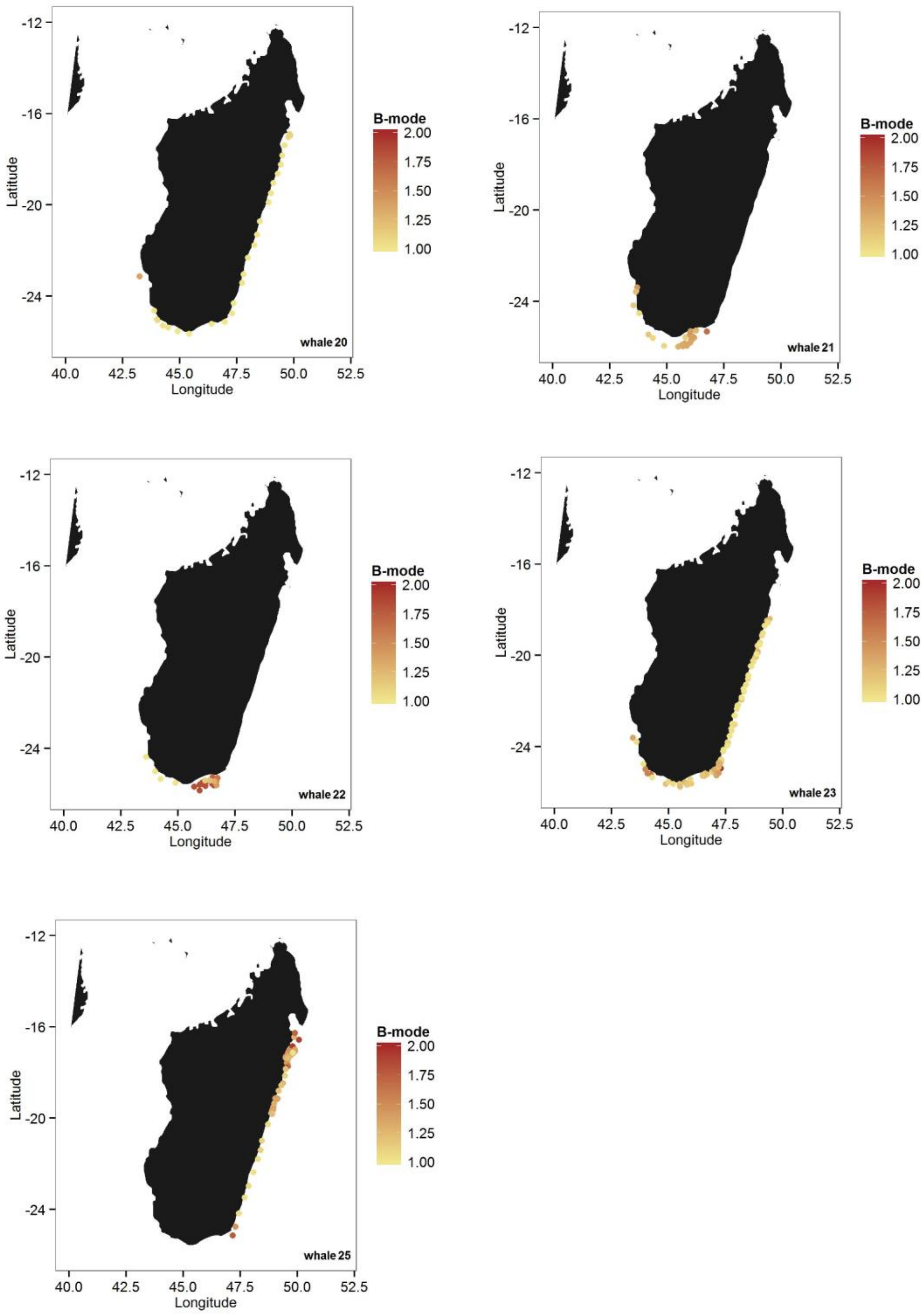

Figure S4b. Maps of female coastal movements showing b-mode values for each estimated location. It ranges from 1, meaning low probability of localized movement and 2 meaning high probability of localized movement. In alphabetical order: whale 20, whale 21 whale 22, whale 23, and whale 25.

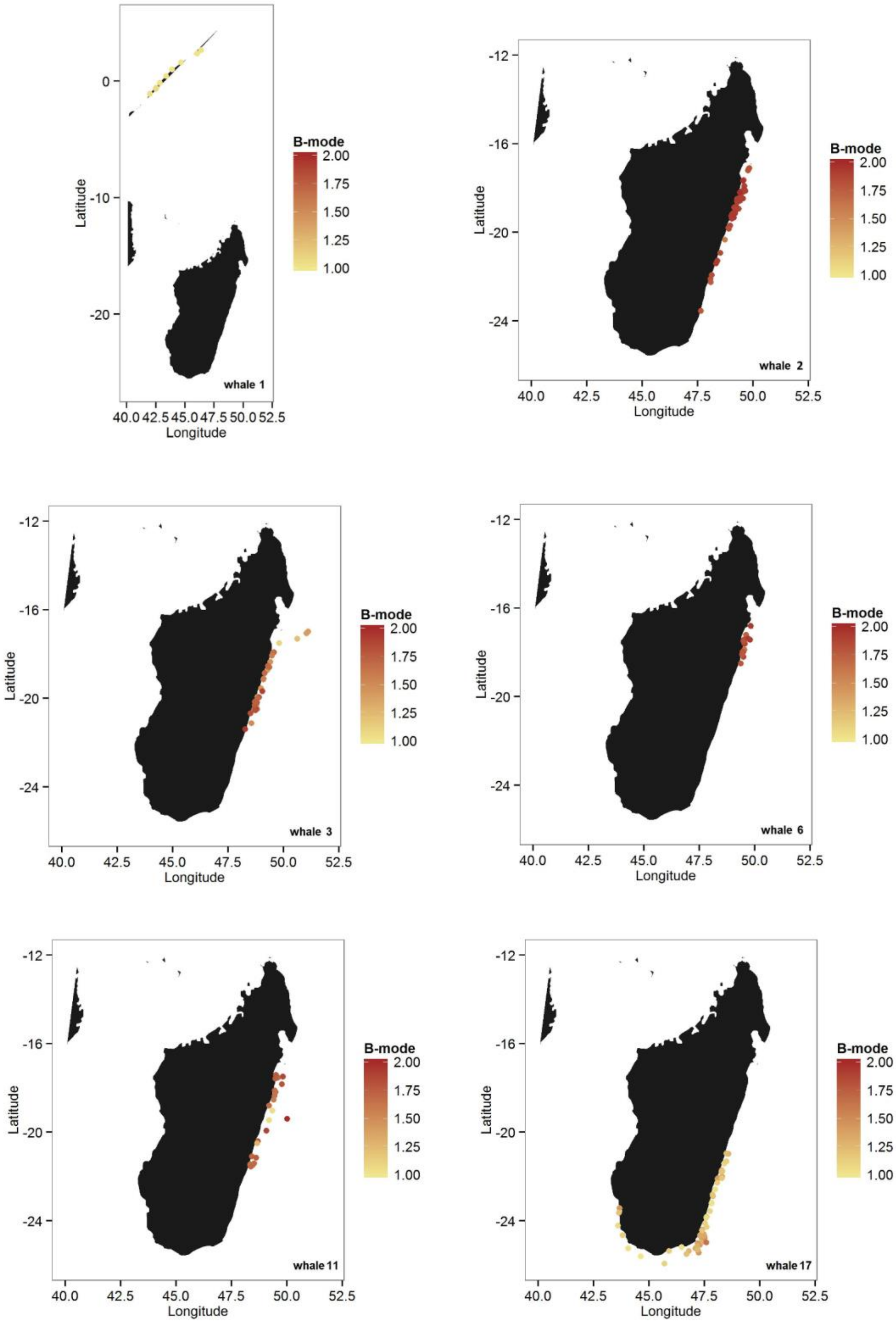

Figure S5a. Male coastal movements showing b-mode values for each estimated location. It ranges from 1, meaning low probability of localized movement and 2 meaning high probability of localized movement. In alphabetical order: whale 1, whale 2, whale 3, whale 6, whale 11, and whale 17.

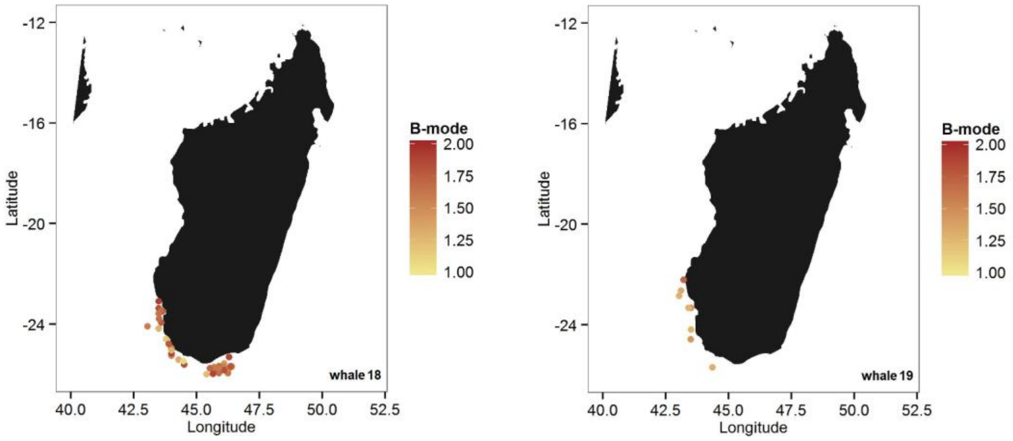

Figure S5b. Male coastal movements showing b-mode values for each estimated location. It ranges from 1, meaning low probability of localized movement and 2 meaning high probability of localized movement. In alphabetical order: whale 18, and whale 19.

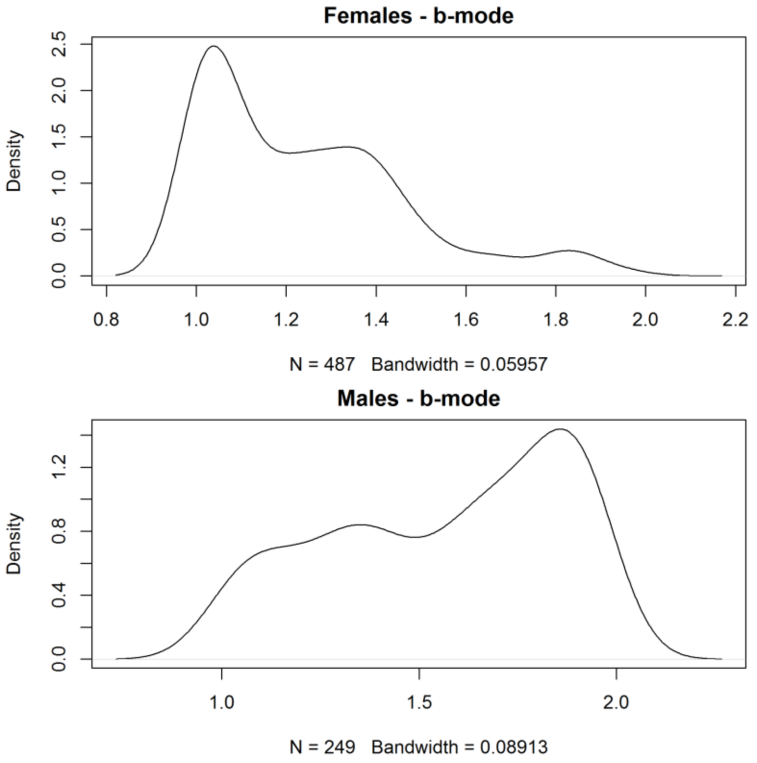

Figure S6. Density distributions of b-mode values for females and males during coastal movements.

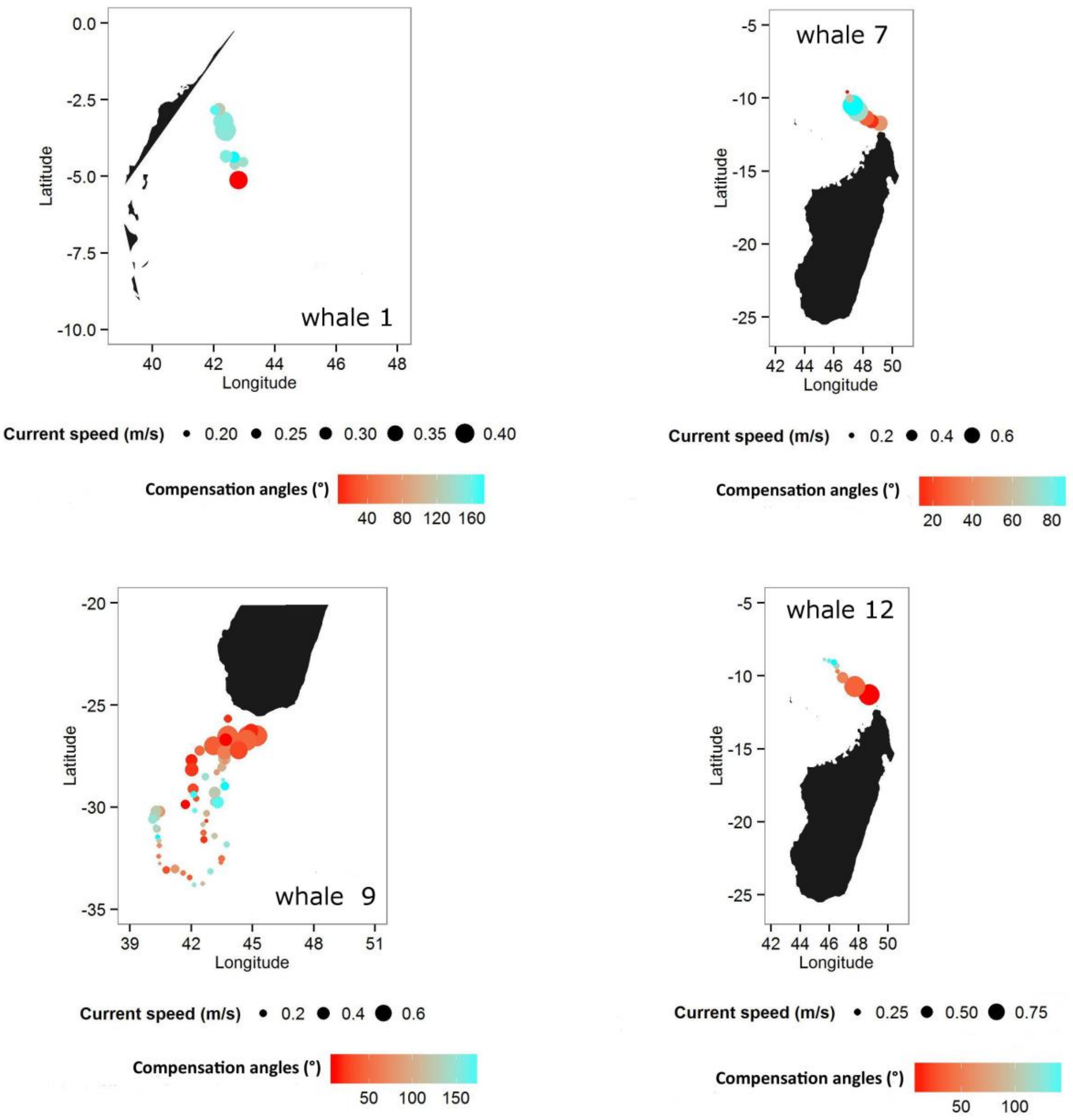

Figure S7a. Current speed for each whale oceanic location of whales 1, 7, 9 and 12. Colour scale shows the compensation angles.

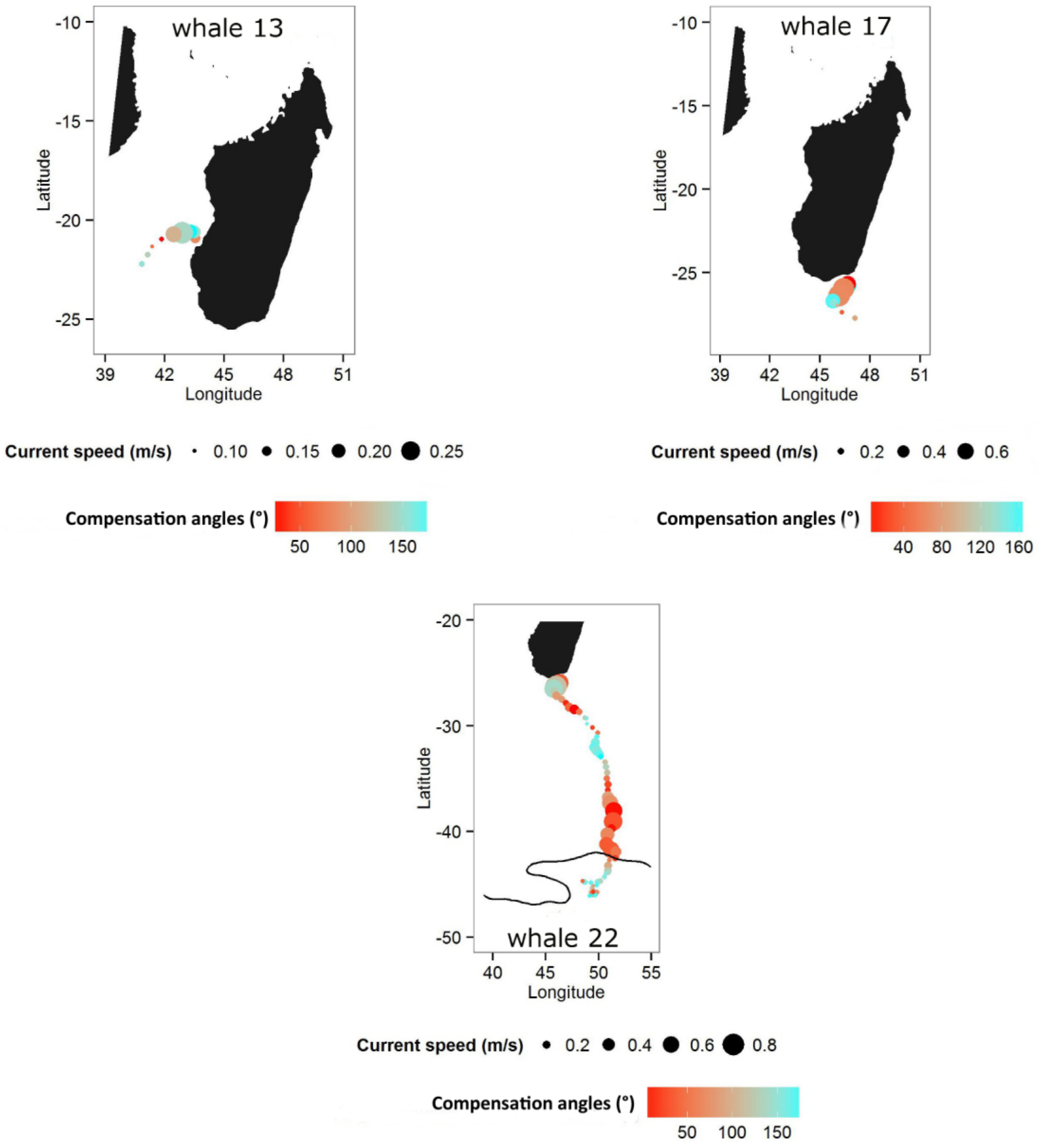

Figure 7b. Current speed for each whale oceanic location of whales 13, 17 and 22. Colour scale shows the compensation angles.

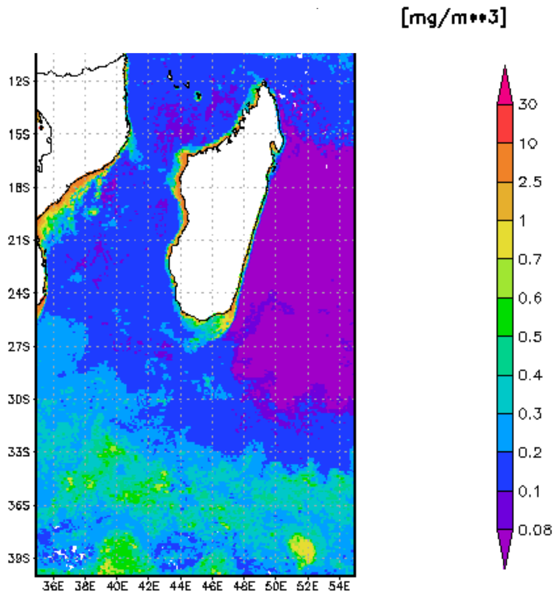

Figure 8. Chlorophyll-a concentration (mg/m^{-3}) in September 2013 on Walters shoal seamount obtained from the NASA AQUA MODIS satellite data (http://disc.sci.gsfc.nasa.gov/giovanni).
